# Supplementary material for: The effects of inhaled corticosteroids on healthy airways
Source: Allergy. Author manuscript; Available in PMC 2024 Jul 10. (PMC7616167; doi:10.1111/all.16146)
Supplement: Online supplement [file EMS194910-supplement-Online_supplement.pdf]

ONLINE SUPPLEMENT

The effects of inhaled corticosteroids on healthy airways

[1]Emanuele Marchi, PhD*, [1]Timothy S.C. Hinks, FRCP, PhD*, [2]Matthew Richardson, PhD, [2]Latifa Khalfaoui[§], PhD, [2]Fiona A. Symon PhD, [3]Poojitha Rajasekar, BSc, [3]Rachel Clifford, PhD, [2]Beverley Hargadon RGN, [4]Cary D. Austin MD, PhD, [5]Julia L. MacIsaac, PhD, [5]Michael S. Kobor, PhD, [2]Salman Siddiqui[§], FRCP, [4]Jordan S. Mar, PhD, [4]Joseph R. Arron MD, PhD, [4]David F. Choy BSc, [2]Peter Bradding FRCP, DM.

[1]NIHR Oxford Respiratory BRC and Respiratory Medicine Unit, Experimental Medicine, Nuffield Department of Medicine, John Radcliffe Hospital, Oxford, UK.

[2] Department of Respiratory Sciences, University of Leicester, Leicester Respiratory NIHR BRC, Glenfield Hospital, Leicester, UK.

[3] Centre for Respiratory Research, Translational Medical Sciences, School of Medicine; Nottingham NIHR Biomedical Research Centre; and Biodiscovery Institute, University Park, University of Nottingham, Nottingham, UK.

[4] Genentech, Inc. South San Francisco, CA, USA.

[5]Edwin S.H. Leong Centre for Healthy Aging, Department of Medical Genetics, University of British Columbia, Vancouver, Canada.

*These authors contributed equally to this manuscript.

Corresponding author

Professor Peter Bradding, Department of Respiratory Sciences, University of Leicester, Glenfield Hospital, Leicester, LE3 9QP, UK. Tel: +44 116 258 3998

E-mail: pb46@le.ac.uk

Running title: The effects of ICS on healthy airways

METHODS

This prospective study was approved by the East Midlands - Leicester Central Research Ethics Committee (REC)(reference 15/EM/0313) and registered at clinicaltrials.gov (NCT02476825). All participants gave written informed consent.

Participant population

People aged 18-65 were eligible, and all were current non-smokers with a <10 pack year smoking history. They had no prior history or clinical evidence of lower respiratory disease, and normal spirometry. Healthy volunteers with a history of rhinitis (perennial or seasonal) were required to have a PC_{20} methacholine >16 mg/ml. Atopy was defined as a positive skin prick test to common aeroallergens.

Participant characterisation

Participants underwent extensive evaluation at baseline including a full medical history, lung function testing, with bronchial challenge using methacholine where appropriate.

Study design

This was a randomised, open-labelled, bronchoscopy study designed to assess the effects of fluticasone propionate on airway gene expression and cellularity in healthy adult healthy volunteers without asthma. The primary endpoint was the corticosteroid-inducible gene expression pattern in healthy airways. Secondary endpoints were the relative changes from baseline in airway cellularity, DNA methylation and the microbiome following 4-weeks of inhaled fluticasone propionate treatment.

Target recruitment was 30 healthy adult subjects randomised by a blinded investigator (MR) in a 2:1 ratio to one of two study groups: i) subjects receiving fluticasone propionate 500 mcg b.i.d. via Accuhaler (Diskus) for 4 weeks (n=20), or ii) subjects who received no treatment for 4 weeks (n=10). Bronchoscopy was performed in all subjects at baseline prior to the start of the treatment period, and

at the end of week 4. Genentech and Leicester laboratory support staff were blinded to treatment allocation. A control arm was included to provide a comparator for the treatment group and to assess the repeatability of the planned analyses.

To ensure there were sufficient data for analysis, if a subject withdrew before completion of the study, a further subject(s) was randomised after the first 30 randomisations until a total of 30 subjects had completed the study.

Bronchoscopy

Subjects underwent bronchoscopy conducted according to British Thoracic Society guidelines (E1). Mucosal biopsies and brushes were collected from 2nd-5th generation bronchi under direct vision as per study procedure manual.

Tissue processing and immunohistochemistry

Biopsies were fixed in 4% neutral buffered formalin for 4 hours at 4°C as described (E2), then processed into paraffin wax, as per study procedure manual. Immunohistochemistry was performed in Leicester. All the laboratory procedures and processes were performed following the ISO9001-2015 Quality Management System and GCP/GLP guidelines.

3 μm tissue sections were cut and dewaxed, and stained with haematoxylin and eosin for quality control assessment, to ensure the integrity of the key tissue elements needed for immunohistochemistry staining and analysis. Further 3 μm sections were immunostained with the following mouse monoclonal primary antibodies: anti–neutrophil elastase (clone NP57, 0.42 μg/mL, Agilent Dako, UK), anti–mast cell tryptase clone AA1 (ready-to-use [RTU], Agilent Dako), anti-mast cell chymase (LS-B12242, 0.8 μg/mL, LSBio, UK), anti–eosinophil major basic protein (clone BMK13, 1 μg/mL, Monosan, UK), anti-α-smooth muscle actin (αSMA)(RTU, Dako) and appropriate isotype controls (Dako and BD Biosciences). All immunostaining steps were performed using an Autostainer Link 48 (Agilent Dako, UK) using appropriate isotype controls followed by counterstaining in Gill's haematoxylin. Two sections from 2 biopsies were stained for each parameter.

Assessment of immunopathology

High-throughput morphologic analysis was performed on scanned sections using the Carl Zeiss Scanner Z1 and AxioCam HRc digital camera (Carl Zeiss, Germany). ZEN desk 3.1 image analysis software was used to perform image analysis on total, ASM, epithelium and lamina propria areas. The following previously validated pathological features (E3) were evaluated as follows; i) nucleated inflammatory cells (eosinophils, neutrophils, mast cells [tryptase+ and chymase+]) were counted in the airway epithelium and lamina propria, and expressed as cells/mm^2 of the compartment of interest; ii) the percentage of biopsy area occupied by airway smooth muscle was measured and expressed as a percentage of the total biopsy area; iii) reticular basement membrane (RBM) thickness was expressed as the mean value of 50-point measurements approximately 20 μm apart according to the method validated by Sullivan *et al* (E4). The mean of two sections from 2 biopsies was taken for each analysis.

All pathological data were assessed by an observer blinded to the identity and treatment allocations of the participants.

RNA sequencing

Bronchial brushes and biopsies were collected in RNAlater as per study procedure manual. Total RNA and DNA separately were isolated from the bronchial brushes and biopsies using QIAgen AllPrep DNA/RNA/miRNA Universal Mini Kit per manufacturer's instructions. Ribosomal RNA was removed using the RiboZero Magnetic Gold kit. RNA sequencing libraries were prepared using the Illumina TruSeq Stranded Total RNA method. Libraries were single-end sequenced. The RNA Sequencing data have been deposited in the Gene Expression Omnibus (GEO) under accession number GSE242048.

Cell deconvolution analysis

Cell deconvolution analysis was performed using MuSiC. Reference cell signatures were derived from single cell data from the Lung Cell Atlas (E5) and annotated with Azimuth in R to assess

predicted changes in cell numbers. Additional gene set enrichment analysis was performed using cell signatures referenced from Travaglini (E6) between day 0 and week 4 of ICS therapy.

To determine whether ICS have differential effects on specific epithelial cell types we used the proportions of 5 different epithelial and structural cell types (multiciliated, basal resting, peribronchial fibroblasts, club and suprabasal), estimated using MuSiC deconvolution, to estimate differential gene expression between treatment conditions according to cell type, using TOAST (E7) in R. Results are presented in **Supplementary table E9**.

Bisulphite conversion and DNA methylation arrays

Seven hundred fifty ng of purified genomic DNA was bisulfite converted using the EZ DNA Methylation Kit (Zymo Research) as per the manufacturer's instructions. Specific incubation conditions for the Illumina Infinium Methylation Assay were used as per the manufacturer's protocol Appendix. Samples were eluted in 12 µl of the provided elution buffer. Bisulfite-converted DNA was assessed for concentration and quality using a NanoDrop^{TM} 8000 Spectrophotometer (Thermo Fisher Scientific), and 160 ng of the conversion product was used for genome-wide DNA methylation quantification at over 850,000 CpG sites using the Illumina Infinium HumanMethylationEPIC BeadChip array, according to the manufacturer's protocols.

DNA methylation data quality control and normalization

Raw data were obtained from GenomeStudio software in IDAT format. The data were analysed using R statistical software (version 4.1.0). Pre-processing and quality control were performed using *minfi* package (version 1.38.0) (E8). The 65 known quality control SNP probes were used to cluster all samples to detect anomalies within samples from the same donor. Probes were excluded from further analysis according to several criteria: first, the 65 SNP probes, second, 84,953 probes represented by less than three beads in at least 5% of samples; third, 1,721 probes that did not pass detection p value threshold ($p<0.05$) in at least 1% of the samples; fourth, 17,323 probes located on X and Y chromosomes; and finally 26,641 probes that overlap single nucleotide polymorphism and 35979

probes that cross-hybridise to multiple regions on the genome (E9). 699,474 probes remained for analysis. Functional normalisation of filtered probes was carried out using *preprocessFunnorm* function in *minfi* package. Batch effect correction was performed on normalised data to correct for chip and sample position bias using *comBat* function in *sva* package (version 3.40.0)(E10). Two values of DNA methylation were calculated, beta-values (β-values) and M-values. β-values are the ratio of all methylated probe intensities over total signal intensities (methylated and unmethylated) and have a range from 0 to 1. They approximately represent percent methylation. M-values are the logit transformation of β-values and are more statistically robust(E11). All statistical analyses were performed using M-values, while β-values were used for visualization and interpretability purposes.

Differential DNA methylation analysis

Linear regression analysis was applied to the data using the *limma* package (version 3.48.0) in R(E12). An interaction term and subject correlation were included to identify any probes differentially methylated between baseline and week 4 in individuals taking fluticasone independently to the control untreated individuals (Methylation~Timepoint + Treatment + TimepointxTreatment + [1 | Subject ID]).

Expression quantitative trait methylation (eQTM) analysis

Association of DNA methylation with differential expression was studied using expression quantitative trait methylation analysis. Twenty nine differentially expressed genes and comprehensive DNA methylation data (699474 probes resulting after probe filtering) from week 0 and week 4 fluticasone treatment samples were given as input into an eQTM linear regression model to identify significantly correlating expression-methylation pairs. Significant pairs were classified based on a Benjamini-Hochberg false discovery rate (FDR) less than 0.05.

Microbiota Sequence Data Generation, Processing, and Analysis

DNA isolated from bronchial brushes was sent to Diversigen for bacterial 16SV4 rRNA gene sequencing as previously described (E13,14). Briefly, PCR amplification of the 16S rRNA gene was

conducted for each sample using barcoded primers targeting the 16S-V4 region. Following PCR, amplicon libraries were pooled at equimolar concentrations and purified. Pooled amplicon sequence libraries were then sequenced with a MiSeq 600 cycle v3 kit (Illumina, San Diego, CA). Following sequencing, QIIME2 v2019.7 was used to process the 16S-V4 rRNA gene sequence data as previously described (E15,16). Briefly, raw sequence data was demultiplexed, read trimming was performed to remove regions of low sequence quality, and paired-end reads were denoised, dereplicated, and chimera filtered with DADA2(E17). The remaining non-chimeric reads were then filtered to remove reads mapping to the human genome using bowtie2 (E18). Taxonomy was assigned based on the V4 region of the Genome Taxonomy Database 16S rRNA gene sequence database (r202) (E19). Resulting abundance tables were rarefied to 8900 reads per sample to account for variable sequencing depth prior to calculation of α- and β-diversity metrics and samples with less than 8900 high quality reads were discarded. All statistical analyses were conducted in the R statistical environment as previously described (Team RC. R: A Language and Environment for Statistical Computing. Vienna, Austria; 2020) (E20). All microbiome sequence data is available from the European Genome-Phenome Archive (https://ega-archive.org/, EGAS00001007538).

Transcriptomic analysis

For analysis of RNA-seq data, sequences in fastq files (in single and pair ends) were aligned using STAR aligner (version 2.7.1a) to the human reference genome GRCh38; R package (E21)*Rsubread* was employed for quantification of reads assigned to genes.

Raw count pre-processing, normalisation and differential gene expression analysis was performed using R, packaged edgeR (for pre-processing and gene expression filtering) and gene expression analysis with *DESeq2*, and *limma*. Gene lists of differentially expressed genes and over-representation analysis of gene pathways/categories were produced from *DESeq2* and *limma* results; volcano plots were generated with log2 fold changes and adjusted p values resulting from moderated t tests in *limma*.(E21,22).

Statistical analysis

Basic summary statistical analysis was performed using GraphPad Prism version 7.03 (GraphPad Software, San Diego). Parametric and non-parametric data are presented as mean (standard error mean [SEM]) and median (interquartile range [IQR]) respectively unless otherwise stated.

SUPPLEMENTARY RESULTS

Comparison with corticosteroid-treated structural cells from published *in vitro* transcriptomic datasets

To compare the effects of corticosteroid treatment on specific structural cells we checked for variations in gene expression reanalysing publicly available data obtained from fibroblast cell lines, primary smooth muscle cells and primary respiratory epithelium treated *in vitro* with corticosteroids. Leung *et al* (E23) analysed the effect of cortisol on the IMR-90 human lung fibroblast cell line. We found no enrichment of this steroid-induced fibroblast geneset, using GSEA on genes stringently up regulated between cortisol and vehicle control, at log fold change > 3 and adjusted $p < 0.05$ when assessing our genes from our dataset differentially expressed between day 0 and week 4 (**Supplementary Figure E12, panel A**).

By contrast we observed very strong enrichment of a geneset derived from primary human airway smooth muscle cells treated with fluticasone propionate, from Jackson *et al* (E24). In this study primary airway smooth muscle cells were dissected from trachea and lung tissue from paediatric patients and treated *ex vivo* with fluticasone propionate (FP). This geneset was highly enriched, normalised enrichment score (NES) 2.7, adjusted $P < 1\text{x}10^{-11}$. (**Supplementary Figure E12, panel B**).

Likewise we observed very strong enrichment of a geneset derived from primary human airway epithelial cells treated *in vitro* with budesonide, from Ginebaugh *et al* (E25) . This geneset was highly enriched, normalised enrichment score (NES) 2.5, adjusted $P < 1\text{x}10^{-11}$. (**Supplementary Figure E12, panel C**).

Effects of ICS on the airway microbiome

To assess the potential impact of ICS treatment on the bacterial community composition of the lower airways, bronchial brushing DNA from paired baseline and week 4 samples from treated and untreaded individuals was subjected to 16SV4 rRNA gene sequencing (16SV4-seq). Processing

resulted in high quality 16SV4-seq data from paired baseline/week 4 samples from 6 untreated and 12 ICS-treated individuals. Between baseline and week 4, no difference in α-diversity was observed, regardless of treatment group. Additionally, no difference in gross bacterial community composition based on β-diversity was observed when comparing baseline and week 4 samples between treated and untreated participants. At the individual taxa level, the relative abundance of only two species was significantly changed (Benjamini-Hochberg adjusted p-value < 0.05) following ICS treatment. Relative abundance of *Alloprevotella tannerae* was significantly increased at week 4 in ICS-treated individuals compared to baseline while that of an unclassified *Corynebacterium* member was decreased (**Supplementary Figure E13**).

Effects of ICS on airway DNA methylation

Linear regression analysis identified only a single significantly differentially methylated CpG site, cg14383238 (Benjamini Hochberg p=0.013) (**Supplementary Figure E14**), suggesting global airway epithelial cell DNA methylation is minimally affected by short term fluticasone treatment. cg14383238 lies on north shore of a CpG island located in the promoter associated transcription start site of transmembrane protein 63C (TMEM63C) located on chromosome 14. This gene is associated with Ca^{2+}-activated cation channel activity required for kidney glomerular filtration barrier function.

Having identified 29 differentially expressed genes in response to four weeks inhaled corticosteroid treatment in bronchial epithelial cells, we sought to investigate the involvement of DNA methylation in regulating these changes. Expression quantitative trait methylation (eQTM) analysis was performed using differentially expressed genes and 699474 CpG sites in matched samples of bronchial brushing derived epithelial cells. There were 48978 significant gene-CpG correlations (Benjamini Hochberg p<0.05) that comprised 29 differentially expressed genes and 30389 CpG sites. Despite the correlation, there was no significant difference relative to baseline in the expression-associated CpG sites following 4 weeks of fluticasone treatment.

SUPPLEMENTARY REFERENCES

E1. Du Rand IA, Blaikley J, Booton R, et al. British Thoracic Society guideline for diagnostic flexible bronchoscopy in adults: accredited by NICE. *Thorax* 2013; 68 Suppl 1: i1-i44.

E2. Austin CD, Gonzalez Edick M, Ferrando RE, et al. A randomized, placebo-controlled trial evaluating effects of lebrikizumab on airway eosinophilic inflammation and remodelling in uncontrolled asthma (CLAVIER). *Clin Exp Allergy* 2020; 50: 1342-1351.

E3. Siddiqui S, Shikotra A, Richardson M, et al. Airway pathological heterogeneity in asthma: Visualization of disease microclusters using topological data analysis. *J Allergy Clin Immunol* 2018; 142: 1457-1468.

E4. Sullivan P, Stephens D, Ansari T, Costello J, Jeffery P. Variation in the measurements of basement membrane thickness and inflammatory cell number in bronchial biopsies. *Eur Respir J* 1998; 12: 811-815.

E5. Vieira Braga FA, Kar G, Berg M, et al. A cellular census of human lungs identifies novel cell states in health and in asthma. *Nat Med* 2019; 25: 1153-1163.

E6. Travaglini KJ, Nabhan AN, Penland L, et al. A molecular cell atlas of the human lung from single-cell RNA sequencing. *Nature* 2020; 587: 619-625.

E7. Li Z, Wu H. TOAST: improving reference-free cell composition estimation by cross-cell type differential analysis. *Genome Biol* 2019; 20: 190.

E8. Aryee MJ, Jaffe AE, Corrada-Bravo H, et al. Minfi: a flexible and comprehensive Bioconductor package for the analysis of Infinium DNA methylation microarrays. *Bioinformatics* 2014; 30: 1363-1369.

E9. Pidsley R, Zotenko E, Peters TJ, et al. Critical evaluation of the Illumina MethylationEPIC BeadChip microarray for whole-genome DNA methylation profiling. *Genome Biol* 2016; 17: 208.

E10. Leek JT, Johnson WE, Parker HS, Jaffe AE, Storey JD. The sva package for removing batch effects and other unwanted variation in high-throughput experiments. *Bioinformatics* 2012; 28: 882-883.

E11. Du P, Zhang X, Huang CC, et al. Comparison of Beta-value and M-value methods for quantifying methylation levels by microarray analysis. *BMC bioinformatics* 2010; 11: 587.

E12. Ritchie ME, Phipson B, Wu D, et al. limma powers differential expression analyses for RNA-sequencing and microarray studies. *Nucleic Acids Res* 2015; 43: e47.

E13. Gohl DM, Vangay P, Garbe J, et al. Systematic improvement of amplicon marker gene methods for increased accuracy in microbiome studies. *Nat Biotechnol* 2016; 34: 942-949.

E14. Caporaso JG, Lauber CL, Walters WA, et al. Ultra-high-throughput microbial community analysis on the Illumina HiSeq and MiSeq platforms. *The ISME journal* 2012; 6: 1621-1624.

E15. Wagner F, Mansfield JC, Lekkerkerker AN, et al. Dose escalation randomised study of efmarodocokin alfa in healthy volunteers and patients with ulcerative colitis. *Gut* 2023.

E16. Bolyen E, Rideout JR, Dillon MR, et al. Reproducible, interactive, scalable and extensible microbiome data science using QIIME 2. *Nat Biotechnol* 2019; 37: 852-857.

E17. Callahan BJ, McMurdie PJ, Rosen MJ, Han AW, Johnson AJ, Holmes SP. DADA2: High-resolution sample inference from Illumina amplicon data. *Nat Methods* 2016; 13: 581-583.

E18. Langmead B, Salzberg SL. Fast gapped-read alignment with Bowtie 2. *Nat Methods* 2012; 9: 357-359.

E19. Parks DH, Chuvochina M, Chaumeil PA, Rinke C, Mussig AJ, Hugenholtz P. A complete domain-to-species taxonomy for Bacteria and Archaea. *Nat Biotechnol* 2020; 38: 1079-1086.

E20. Mar JS, Ota N, Pokorzynski ND, et al. IL-22 alters gut microbiota composition and function to increase aryl hydrocarbon receptor activity in mice and humans. *Microbiome* 2023; 11: 47.

E21. Foundation TR. The R Project for Statistical Computing. [cited 2023 23/05/2023]. Available from: http://www.r-project.org/index.html.

E22. Reimers M, Carey VJ. Bioconductor: an open source framework for bioinformatics and computational biology. *Methods Enzymol* 2006; 411: 119-134.

E23. Leung CS, Kosyk O, Welter EM, Dietrich N, Archer TK, Zannas AS. Chronic stress-driven glucocorticoid receptor activation programs key cell phenotypes and functional epigenomic patterns in human fibroblasts. *iScience* 2022; 25: 104960.

E24. Jackson D, Walum J, Banerjee P, et al. Th1 cytokines synergize to change gene expression and promote corticosteroid insensitivity in pediatric airway smooth muscle. *Respir Res* 2022; 23: 126.

E25. Ginebaugh SP, Hagner M, Ray A, et al. Bronchial epithelial cell transcriptional responses to inhaled corticosteroids dictate severe asthmatic outcomes. *J Allergy Clin Immunol* 2023; 151: 1513-1524.
E26. Woodruff PG, Boushey HA, Dolganov GM, et al. Genome-wide profiling identifies epithelial cell genes associated with asthma and with treatment response to corticosteroids. *Proc Natl Acad Sci U S A* 2007; 104: 15858-15863.

Supplementary Table E1. All upregulated differentially expressed genes in bronchial brushes after 4 weeks of inhaled fluticasone (treatment group only). Log2 fold changes and statistical tests calculated using Wald tests in *DESeq2*. Genes shown are censored at FDR P < 0.05 and log(2) fold change of ±1 and ordered by P value. FDR, false discovery rate.

Genes upregulated in bronchial brushes

**Gene**	**Baseline read count**	**Log2 fold change**	**t**	***P* value**	**FDR *P* value**
PHACTR3	497	3.59	14.22	7.3E-46	1E-41
HSD11B2	468	1.89	14.11	3.3E-45	3E-41
MYLK3	476	1.10	13.99	1.8E-44	1E-40
FKBP5	3336	1.88	11.56	6.7E-31	2E-27
BTNL9	369	1.71	11.52	1.1E-30	3E-27
GRAMD2A	2452	1.15	10.81	3E-27	5E-24
IFITM10	1872	1.55	10.36	3.9E-25	4E-22
FAM107A	3486	1.45	10.32	6E-25	6E-22
HCAR2	4673	1.10	9.81	1E-22	9E-20
TSC22D3	7099	1.12	9.28	1.7E-20	1E-17
SLCO1B3	124	1.94	9.24	2.5E-20	2E-17
SULT2B1	1706	1.38	9.17	4.7E-20	3E-17
HIF3A	584	3.18	9.13	7E-20	4E-17

ADAMTS9	204	1.38	9.08	1.1E-19	6E-17
CYP17A1-AS1	115	1.83	9.04	1.5E-19	9E-17
ITGA10	160	1.41	8.73	2.5E-18	1E-15
GP2	341	2.48	8.74	2.4E-18	1E-15
KCNB1	602	1.03	8.68	3.8E-18	2E-15
SYT8	503	1.12	8.62	6.6E-18	3E-15
CD163	1860	2.45	8.51	1.8E-17	7E-15
ANPEP	2159	1.16	8.29	1.1E-16	4E-14
PRODH	5268	1.20	8.26	1.5E-16	6E-14
PSCA	23905	1.21	8.19	2.6E-16	9E-14
TFCP2L1	3228	1.38	8.06	7.9E-16	3E-13
GAS6-AS1	121	1.42	7.86	4E-15	1E-12
GNMT	704	1.28	7.77	7.9E-15	2E-12
TAT-AS1	242	1.01	7.58	3.4E-14	8E-12
ANGPT1	188	1.22	7.41	1.3E-13	3E-11
ABCC3	882	1.00	7.23	4.7E-13	9E-11
PPP1R16B	2159	1.11	7.11	1.2E-12	2E-10
KCNMA1	369	1.12	6.99	2.8E-12	5E-10
GAS1	136	1.15	6.80	1E-11	1E-09
LPL	369	2.41	6.65	2.8E-11	4E-09
LINC00930	204	1.11	6.50	7.9E-11	9E-09
FLVCR2	173	1.01	6.41	1.5E-10	2E-08

MUC21	134	1.51	6.28	3.5E-10	3E-08
HAP1	132	1.17	6.21	5.3E-10	5E-08
VSIG4	808	1.79	5.98	2.3E-09	2E-07
MMP7	203	1.09	5.95	2.7E-09	2E-07
ADAMTSL4	232	1.07	5.80	6.5E-09	4E-07
CPM	202	1.30	5.73	1E-08	6E-07
ERVS71-1	1059	1.51	5.72	1E-08	6E-07
ZBTB16	362	1.88	5.70	1.2E-08	7E-07
MMP19	193	2.05	5.66	1.5E-08	8E-07
GDNF-AS1	119	1.02	5.58	2.4E-08	1E-06
MTUS2	198	1.03	5.31	1.1E-07	5E-06
MS4A4A	231	1.19	5.14	2.7E-07	1E-05
CYP4A11	175	1.34	5.10	3.4E-07	1E-05
GLDN	248	1.17	5.10	3.4E-07	1E-05
SLC11A1	627	1.32	4.73	2.3E-06	6E-05
AOC3	320	1.67	4.72	2.4E-06	6E-05
MME	291	1.44	4.65	3.4E-06	8E-05
LILRA6	117	1.08	4.08	4.4E-05	0.0007
TREM1	436	1.26	3.90	9.5E-05	0.0013
FABP4	1683	1.49	3.86	0.00011	0.0014
TRNA	293	1.30	3.80	0.00015	0.0018
IL1R2	259	1.45	3.73	0.00019	0.0022

SERPINA3	417	1.77	3.71	0.00021	0.0023
KRT6A	1428	1.25	3.48	0.00049	0.0046
CLEC4E	156	1.34	3.29	0.00101	0.0081
ELOA3BP	160	1.01	3.28	0.00104	0.0083
MARCO	1414	1.26	3.28	0.00104	0.0083
MSR1	1008	1.06	3.18	0.00147	0.01
TRNE	9170	1.08	3.17	0.00151	0.01
PCOLCE2	105	1.04	3.12	0.00184	0.01
MRC1	381	1.06	3.11	0.00189	0.01
MCEMP1	186	1.01	2.95	0.00313	0.02
RBP4	164	1.18	2.86	0.00426	0.02
APOC1	1804	1.05	2.75	0.00597	0.03
CXCL13	101	1.86	2.64	0.00838	0.04
APOC1P1	124	1.16	2.63	0.00866	0.041
SAA1	23140	1.34	2.58	0.00992	0.045

Supplementary Table E2. All downregulated differentially expressed genes in bronchial brushes after 4 weeks of inhaled fluticasone (treatment group only). Log2 fold changes and statistical tests calculated using Wald tests in *DESeq2*. Genes shown are censored at FDR P < 0.05 and log(2) fold change of ±1 and ordered by P value. FDR, false discovery rate.

Genes downregulated in bronchial brushes

**Gene**	**Baseline read count**	**Log2 fold change**	**t**	***P* value**	**FDR *P* value**
FCER1A	144	-2.78	-10.91	1.019E-27	2.15E-24
ZNF683 (Hobit)	136	-3.33	-10.66	1.544E-26	2.31E-23
CD207 (Langerin)	169	-3.78	-10.63	2.193E-26	3.05E-23
IL33	3885	-1.20	-10.43	1.792E-25	2.18E-22
CLEC4F	102	-2.18	-10.35	4.237E-25	4.59E-22
CCL5 (RANTES)	1133	-2.39	-8.74	2.232E-18	1.21E-15
PTGS1	129	-1.19	-8.64	5.515E-18	2.5E-15
HEPACAM2	450	-1.00	-8.59	8.478E-18	3.67E-15
TARP	100	-2.50	-8.51	1.724E-17	7.3E-15
CD96	253	-1.62	-7.85	4.091E-15	1.31E-12
ABCB11	147	-1.32	-7.52	5.653E-14	1.33E-11
UCN3	239	-1.05	-7.30	2.792E-13	5.67E-11

TRBC1	353	-2.33	-7.04	1.95E-12	3.39E-10
CX3CR1	102	-1.49	-7.04	1.969E-12	3.4E-10
TRBC2	463	-2.41	-6.92	4.595E-12	7.16E-10
CD3E	375	-1.55	-6.87	6.523E-12	9.48E-10
PTPN22	165	-1.26	-6.80	1.017E-11	1.43E-09
TRAC	539	-1.92	-6.67	2.559E-11	3.37E-09
IGF2BP3	140	-1.29	-6.66	2.77E-11	3.6E-09
KLRB1	131	-1.69	-6.65	2.935E-11	3.76E-09
TRAF3IP3	231	-1.01	-6.57	5.098E-11	6.37E-09
CPA3	228	-2.26	-6.55	5.716E-11	7E-09
GIMAP7	143	-1.71	-6.53	6.429E-11	7.68E-09
CD2	566	-1.87	-6.45	1.141E-10	1.23E-08
CLEC10A	135	-1.71	-6.38	1.776E-10	1.82E-08
SERPINB10	287	-1.18	-6.38	1.797E-10	1.82E-08
SCML4	123	-1.09	-6.30	2.994E-10	2.86E-08
TMC8	344	-1.00	-6.22	4.86E-10	4.36E-08
CTSW	148	-1.42	-6.18	6.362E-10	5.46E-08
KLRC1	127	-1.34	-6.12	9.415E-10	7.84E-08
RASAL3	240	-1.06	-6.11	9.794E-10	8.05E-08
MUC13	4313	-1.67	-6.10	1.052E-09	8.58E-08
HSPA7	253	-1.30	-6.06	1.334E-09	1.05E-07
GIMAP6	201	-1.55	-6.02	1.756E-09	1.33E-07

LCK	324	-1.15	-5.99	2.16E-09	1.57E-07
SPOCK2	418	-1.29	-5.77	7.949E-09	4.86E-07
CD8A	516	-1.55	-5.71	1.11E-08	6.4E-07
CD8B	171	-1.26	-5.67	1.418E-08	7.98E-07
PLG	110	-1.08	-5.62	1.912E-08	1.05E-06
ITGA1	273	-1.17	-5.61	2.031E-08	1.11E-06
ITK	119	-1.48	-5.59	2.228E-08	1.2E-06
ITLN1	197	-1.93	-5.52	3.433E-08	1.71E-06
GFI1	133	-1.06	-5.52	3.478E-08	1.73E-06
CD247	132	-1.56	-5.32	1.042E-07	4.61E-06
CD3D	283	-1.66	-5.31	1.1E-07	4.83E-06
RGS7BP	172	-1.06	-5.25	1.505E-07	6.32E-06
KLRC2	106	-1.23	-5.20	2.006E-07	7.95E-06
HLA-DQB2	798	-1.01	-5.16	2.481E-07	9.52E-06
GLYATL2	190	-1.42	-5.06	4.117E-07	1.46E-05
IKZF3	405	-1.35	-5.05	4.425E-07	1.56E-05
CXCR6	396	-1.65	-5.03	4.95E-07	1.72E-05
GIMAP8	116	-1.21	-5.02	5.168E-07	1.78E-05
IGKC	400	-2.29	-4.91	8.899E-07	2.79E-05
IGHG2	175	-2.35	-4.72	2.315E-06	6.23E-05
GVINP1	212	-1.01	-4.66	3.152E-06	8E-05
CCR5	295	-1.42	-4.61	4.078E-06	9.97E-05

LTB	103	-1.98	-4.60	4.236E-06	0.0001
MGAM	455	-1.40	-4.60	4.249E-06	0.0001
TPSAB1	442	-1.65	-4.60	4.31E-06	0.0001
TPSB2	504	-1.66	-4.57	4.81E-06	0.0001
IGHG3	144	-2.34	-4.56	5.064E-06	0.0001
IGHA1	502	-2.01	-4.44	8.875E-06	0.0002
MYO7A	120	-1.00	-4.41	1.014E-05	0.0002
IGHG1	227	-2.24	-4.37	1.242E-05	0.0003
IGLC2	269	-2.33	-4.35	1.335E-05	0.0003
ADAM19	191	-2.03	-4.34	1.42E-05	0.0003
PTPN7	248	-1.32	-4.29	1.787E-05	0.0003
ENPP2	115	-1.09	-4.05	5.074E-05	0.0008
GPR18	137	-1.50	-4.05	5.228E-05	0.0008
NKG7	192	-1.66	-3.92	8.676E-05	0.001
HDAC9	510	-1.20	-3.73	0.0002	0.002
GZMH	137	-1.51	-3.67	0.0002	0.003
UBD	2630	-1.40	-3.62	0.0003	0.003
CD48	271	-1.14	-3.57	0.0004	0.004
IGHA2	201	-1.98	-3.54	0.0004	0.004
IGLC3	216	-2.28	-3.49	0.0005	0.005
MUC2	2840	-1.12	-3.48	0.0005	0.005
FGL2	781	-1.10	-3.38	0.0007	0.006

CD7	187	-1.23	-3.36	0.0008	0.007
CEACAM5	3288	-1.24	-3.36	0.0008	0.007
SLC5A8	1538	-1.01	-3.04	0.002	0.02
IL32	581	-1.09	-2.85	0.004	0.02

Supplementary Table E3. All upregulated differentially expressed genes in bronchial biopsies after 4 weeks of inhaled fluticasone (treatment group only). Log2 fold changes and statistical tests calculated using Wald tests in *DESeq2*. Genes shown are censored at FDR P < 0.05 and log(2) fold change of ±1 and ordered by P value. FDR, false discovery rate.

Genes upregulated in bronchial biopsies

**Gene**	**Baseline read count**	**Log2 fold change**	**t**	***P* value**	**FDR *P* value**
PHACTR3	325	3.28	13.65	2.082E-42	4.287E-38
HSD11B2	392	1.81	13.37	9.595E-41	9.879E-37
GP2	286	3.12	11.67	1.906E-31	9.81E-28
SYT8	470	1.13	10.52	6.904E-26	2.37E-22
HIF3A	990	2.67	10.21	1.818E-24	5.347E-21
FKBP5	5029	1.67	10.00	1.526E-23	3.142E-20
FAM107A	3657	1.60	9.22	2.932E-20	4.024E-17
MYLK3	490	1.47	9.16	5.081E-20	6.54E-17
TFCP2L1	4849	1.40	9.06	1.357E-19	1.643E-16
SULT2B1	1199	1.44	8.73	2.441E-18	2.011E-15
GRAMD2A	2309	1.38	8.70	3.398E-18	2.691E-15
GMPR	694	1.09	8.61	7.136E-18	4.592E-15
HCAR3	1622	1.10	8.40	4.361E-17	2.566E-14

PSCA	13008	1.87	8.35	7.063E-17	3.828E-14
SLCO1B3	111	2.27	8.31	9.972E-17	5.106E-14
HCAR2	3361	1.22	8.20	2.505E-16	1.146E-13
ATP6V1C2	1007	1.24	8.03	9.549E-16	4.013E-13
PRODH	4754	1.10	7.94	2.075E-15	8.217E-13
TSC22D3	8967	1.14	7.84	4.357E-15	1.547E-12
PDK4	2453	1.27	7.44	1.031E-13	2.869E-11
IFITM10	1816	1.23	7.40	1.407E-13	3.713E-11
ANPEP	1707	1.02	7.31	2.592E-13	6.28E-11
KCNB1	662	1.28	6.97	3.25E-12	6.626E-10
ERVS71-1	810	2.02	6.70	2.045E-11	3.29E-09
NPTX2	109	1.63	6.70	2.15E-11	3.405E-09
PAQR4	943	1.02	6.59	4.3E-11	5.982E-09
PKIB	2384	1.21	6.34	2.364E-10	2.735E-08
GNMT	548	1.10	5.97	2.427E-09	1.968E-07
TAT-AS1	146	1.10	5.82	5.916E-09	4.094E-07
AACSP1	109	1.29	5.48	4.197E-08	2.199E-06
SEC14L5	584	1.08	5.38	7.27E-08	3.506E-06
GALNT15	461	2.29	4.55	5.267E-06	0.0001
CLDN8	1164	1.16	4.52	6.297E-06	0.0002
MTUS2	196	1.30	4.48	7.342E-06	0.0002
FOXN4	222	1.03	4.47	7.923E-06	0.0002

CDC20B	903	1.15	4.45	8.653E-06	0.0002
LINC00964	534	1.13	4.30	1.738E-05	0.0004
KLHDC8A	405	1.21	4.25	2.108E-05	0.0005
MUC21	117	1.36	4.24	2.23E-05	0.0005
SLC6A1	179	1.30	3.80	0.0001423	0.0023
ZBTB16	759	1.11	3.79	0.0001514	0.002
SERPINA3	1487	1.41	3.68	0.0002316	0.003
APOD	6137	1.09	3.64	0.0002693	0.004
CD163	887	1.06	3.50	0.0004666	0.006
PRH2	3255	1.52	3.15	0.0016383	0.02
H1-3	144	1.02	3.05	0.0022815	0.02
PRB3	26511	1.47	2.98	0.0028657	0.03
FOSB	812	1.46	2.97	0.0029993	0.03
SAA1	8478	1.09	2.96	0.0030546	0.03
COMP	185	1.18	2.91	0.0036188	0.03
PRB1	6653	1.55	2.88	0.0039479	0.03
PRB4	15992	1.59	2.85	0.0043256	0.03
PRB2	6775	1.53	2.85	0.0043641	0.03

Supplementary Table E4. All downregulated differentially expressed genes in bronchial biopsies after 4 weeks of inhaled fluticasone (treatment group only). Log2 fold changes and statistical tests calculated using Wald tests in *DESeq2*. Genes shown are censored at FDR P < 0.05 and log2 fold change of ±1 and ordered by P value. FDR, false discovery rate.

**Genes downregulated in bronchial biopsies**					
**Gene**	**Baseline read count**	**Log2 fold change**	**t**	***P* value**	**FDR *P* value**
FCER1A	228	-2.25	-11.91	1.01E-32	6.93E-29
ZNF683 (Hobit)	131	-3.06	-10.10	5.78E-24	1.32E-20
CD207 (Langerin)	144	-2.90	-9.48	2.47E-21	4.24E-18
HLA-DQB2	640	-1.51	-9.47	2.92E-21	4.63E-18
TNFSF15	212	-1.56	-9.29	1.58E-20	2.32E-17
CCL5 (RANTES)	1233	-2.15	-8.98	2.72E-19	2.67E-16
IL33	6985	-1.24	-8.66	4.85E-18	3.70E-15
P4HA3	105	-1.72	-8.64	5.40E-18	3.83E-15
DPP4	481	-1.02	-8.62	6.91E-18	4.59E-15
CD96	399	-1.64	-8.36	6.27E-17	3.49E-14
THEMIS	146	-1.71	-8.36	6.14E-17	3.49E-14
CLEC10A	199	-2.20	-8.33	8.31E-17	4.39E-14

CD8A	611	-1.52	-8.30	1.02E-16	5.11E-14
CPVL	624	-1.16	-8.25	1.61E-16	7.91E-14
TRGC2	103	-1.98	-8.23	1.93E-16	9.23E-14
CD2	732	-1.87	-8.22	2.05E-16	9.60E-14
TRBC2	741	-2.07	-8.08	6.67E-16	2.98E-13
TRBC1	540	-2.07	-8.05	8.33E-16	3.65E-13
TRAC	764	-1.84	-8.02	1.09E-15	4.48E-13
DOCK10	713	-1.55	-7.90	2.77E-15	1.07E-12
CCR5	426	-1.91	-7.90	2.90E-15	1.11E-12
SERPINB10	350	-1.42	-7.88	3.41E-15	1.25E-12
CD3G	114	-1.74	-7.82	5.49E-15	1.91E-12
UBASH3A	123	-1.92	-7.78	7.42E-15	2.55E-12
NA	131	-3.10	-7.71	1.29E-14	4.27E-12
P2RY10	115	-1.90	-7.64	2.15E-14	6.71E-12
SPN	381	-1.55	-7.61	2.65E-14	8.01E-12
PIK3CG	263	-1.59	-7.34	2.17E-13	5.45E-11
PTPN22	243	-1.52	-7.33	2.32E-13	5.76E-11
RUFY4	117	-1.54	-7.28	3.44E-13	8.04E-11
NAPSB	333	-1.52	-7.20	5.94E-13	1.38E-10
GPRIN3	376	-1.60	-7.18	7.17E-13	1.62E-10
PTPN7	312	-1.64	-7.12	1.07E-12	2.36E-10
GFI1	177	-1.23	-7.11	1.14E-12	2.46E-10

DNASE1L3	156	-1.79	-7.10	1.29E-12	2.76E-10
CCR6	142	-1.27	-7.09	1.30E-12	2.76E-10
TARP	112	-1.90	-7.07	1.52E-12	3.18E-10
IGHV1-46	241	-2.93	-7.00	2.62E-12	5.40E-10
IGLV1-50	195	-2.49	-6.96	3.38E-12	6.83E-10
ITGB7	427	-1.12	-6.85	7.46E-12	1.45E-09
CPA3	913	-1.98	-6.84	7.90E-12	1.51E-09
CD8B	206	-1.38	-6.79	1.09E-11	1.99E-09
LCK	441	-1.38	-6.78	1.17E-11	2.11E-09
KLRC2	101	-1.29	-6.77	1.26E-11	2.24E-09
FPR3	291	-1.65	-6.77	1.28E-11	2.26E-09
FCGR2B	224	-1.57	-6.76	1.34E-11	2.31E-09
ELK2AP	1234	-3.29	-6.76	1.33E-11	2.31E-09
HLA-DPB2	156	-1.28	-6.75	1.45E-11	2.49E-09
ZNF831	150	-1.54	-6.74	1.56E-11	2.66E-09
LY9	150	-1.23	-6.74	1.58E-11	2.67E-09
TLR7	127	-1.47	-6.72	1.83E-11	2.99E-09
COL6A5	136	-3.00	-6.68	2.45E-11	3.83E-09
APLNR	328	-3.17	-6.67	2.49E-11	3.86E-09
IGKV1D-8	209	-2.99	-6.67	2.54E-11	3.87E-09
CD3D	291	-1.64	-6.66	2.74E-11	4.15E-09
CXCR6	432	-1.71	-6.66	2.82E-11	4.21E-09

IGHG4	4984	-3.20	-6.65	2.85E-11	4.22E-09
IGHV3OR16-8	106	-3.11	-6.65	3.03E-11	4.43E-09
CX3CR1	241	-1.30	-6.60	4.22E-11	5.96E-09
HLA-DPB1	5797	-1.25	-6.58	4.73E-11	6.46E-09
IKZF3	711	-1.39	-6.56	5.45E-11	7.33E-09
IGKV1D-17	284	-3.12	-6.55	5.83E-11	7.79E-09
IGKV2-40	197	-2.98	-6.54	6.18E-11	8.21E-09
LOC642131	282	-2.41	-6.46	1.04E-10	1.32E-08
IGHV4-4	204	-2.84	-6.46	1.05E-10	1.33E-08
IGHV3-48	573	-2.85	-6.44	1.19E-10	1.50E-08
LOC102724760	122	-2.23	-6.43	1.25E-10	1.55E-08
HDAC9	686	-1.14	-6.42	1.39E-10	1.72E-08
IGHV3-11	470	-3.00	-6.41	1.47E-10	1.81E-08
HLA-DQA2	1904	-1.36	-6.40	1.51E-10	1.85E-08
IGKV4-1	807	-3.16	-6.39	1.67E-10	2.03E-08
IGKV1-8	247	-3.17	-6.39	1.71E-10	2.04E-08
CCR2	290	-1.84	-6.39	1.70E-10	2.04E-08
AKAP5	108	-1.01	-6.38	1.74E-10	2.07E-08
LAX1	112	-1.59	-6.34	2.26E-10	2.63E-08
NA	120	-3.05	-6.33	2.44E-10	2.80E-08
IGKV1-16	430	-3.31	-6.33	2.52E-10	2.88E-08
IGHJ6	119	-2.63	-6.32	2.61E-10	2.94E-08

SAMSN1	213	-1.51	-6.32	2.64E-10	2.96E-08
ITGA4	748	-1.02	-6.28	3.38E-10	3.67E-08
IGHV3-35	105	-2.78	-6.25	4.02E-10	4.29E-08
MYO7A	150	-1.33	-6.25	4.11E-10	4.37E-08
SOSTDC1	219	-1.12	-6.25	4.22E-10	4.46E-08
SLAMF1	162	-1.78	-6.24	4.33E-10	4.55E-08
HLA-DQA1	3663	-1.31	-6.23	4.74E-10	4.93E-08
HLA-DQB1	4235	-1.04	-6.22	5.10E-10	5.22E-08
CD3E	601	-1.38	-6.21	5.34E-10	5.45E-08
IGLV1-40	629	-2.83	-6.21	5.41E-10	5.49E-08
IGHV4-55	245	-2.58	-6.20	5.79E-10	5.81E-08
LOC102724971	328	-2.49	-6.19	6.07E-10	6.00E-08
MPEG1	1046	-1.51	-6.19	6.15E-10	6.06E-08
KEL	124	-1.45	-6.18	6.22E-10	6.10E-08
IGHV4-31	178	-2.80	-6.18	6.51E-10	6.35E-08
IGHV3-71	152	-2.82	-6.17	7.01E-10	6.75E-08
SCML4	246	-1.30	-6.16	7.14E-10	6.83E-08
SPOCK2	833	-1.32	-6.16	7.29E-10	6.95E-08
IGKV1-6	331	-3.00	-6.15	7.53E-10	7.14E-08
KLRC1	124	-1.20	-6.14	8.12E-10	7.63E-08
IGKV3-7	256	-2.97	-6.11	9.70E-10	9.03E-08
IGHV1-18	424	-3.04	-6.11	1.02E-09	9.44E-08

IGHG3	11442	-3.13	-6.10	1.04E-09	9.57E-08
SASH3	585	-1.44	-6.08	1.19E-09	1.09E-07
AOAH	400	-1.28	-6.07	1.32E-09	1.20E-07
KCNA3	252	-1.80	-6.06	1.33E-09	1.20E-07
CD226	204	-1.21	-6.06	1.33E-09	1.20E-07
THY1	274	-2.18	-6.06	1.39E-09	1.24E-07
IGKV3D-7	660	-2.86	-6.06	1.40E-09	1.24E-07
GRAP2	236	-1.54	-6.05	1.47E-09	1.29E-07
MS4A2	264	-2.20	-6.04	1.52E-09	1.34E-07
IGKV1-13	363	-2.98	-6.02	1.75E-09	1.50E-07
LCP2	550	-1.32	-6.02	1.76E-09	1.51E-07
IGHV3-65	128	-2.87	-6.01	1.80E-09	1.53E-07
IGKV3-15	1137	-3.15	-6.01	1.83E-09	1.54E-07
KLRB1	226	-1.50	-6.00	2.01E-09	1.67E-07
SLC5A7	236	-1.20	-5.98	2.17E-09	1.79E-07
IGHV3-53	279	-2.86	-5.96	2.51E-09	2.02E-07
IGKV1-17	498	-3.15	-5.96	2.59E-09	2.06E-07
IGKV2D-40	196	-3.05	-5.96	2.58E-09	2.06E-07
ZAP70	461	-1.09	-5.95	2.65E-09	2.10E-07
JCHAIN	6687	-2.83	-5.95	2.72E-09	2.14E-07
FAM78A	254	-1.20	-5.94	2.82E-09	2.20E-07
IGHV3-72	126	-2.97	-5.92	3.20E-09	2.47E-07

GPR171	112	-1.39	-5.92	3.23E-09	2.48E-07
IGHV3-41	107	-2.82	-5.90	3.54E-09	2.68E-07
IGKV1D-39	1300	-3.05	-5.89	3.97E-09	2.98E-07
TESPA1	139	-1.81	-5.87	4.30E-09	3.18E-07
IL10RA	654	-1.26	-5.87	4.34E-09	3.19E-07
CD101	120	-1.06	-5.87	4.49E-09	3.25E-07
IGHV3-43	129	-2.72	-5.86	4.53E-09	3.27E-07
RASAL3	411	-1.32	-5.86	4.55E-09	3.27E-07
IGHG2	8611	-3.12	-5.86	4.71E-09	3.34E-07
IGKV2D-29	109	-2.79	-5.86	4.69E-09	3.34E-07
IGLC5	323	-2.34	-5.86	4.70E-09	3.34E-07
CD6	416	-1.03	-5.85	4.96E-09	3.51E-07
IGKV3D-15	876	-3.07	-5.84	5.10E-09	3.59E-07
IL12RB1	133	-1.36	-5.83	5.51E-09	3.85E-07
CYSLTR1	265	-1.27	-5.82	6.06E-09	4.16E-07
IGHV3-16	120	-2.86	-5.81	6.11E-09	4.18E-07
IGHV3-21	634	-2.84	-5.81	6.17E-09	4.18E-07
CD5	222	-1.29	-5.80	6.67E-09	4.44E-07
IGKJ1	167	-2.66	-5.80	6.65E-09	4.44E-07
GPR18	146	-1.62	-5.79	7.19E-09	4.74E-07
IGHJ5	133	-2.75	-5.79	7.24E-09	4.76E-07
IGKV1-9	517	-2.87	-5.78	7.61E-09	4.96E-07

SEMA7A	113	-1.33	-5.77	7.74E-09	5.02E-07
IGHV1-69	436	-2.82	-5.77	8.09E-09	5.22E-07
IRF8	423	-1.53	-5.76	8.40E-09	5.37E-07
IGHV3-66	270	-2.86	-5.75	8.77E-09	5.57E-07
IGHJ4	276	-2.81	-5.74	9.32E-09	5.89E-07
NCKAP1L	586	-1.27	-5.74	9.60E-09	6.02E-07
IGHV2-70	103	-2.79	-5.73	9.81E-09	6.10E-07
PTPRC	2347	-1.50	-5.72	1.07E-08	6.62E-07
IGHG1	20412	-3.19	-5.72	1.08E-08	6.65E-07
IGLV3-10	287	-3.02	-5.71	1.11E-08	6.77E-07
IGHV3-22	137	-3.04	-5.71	1.14E-08	6.94E-07
IL2RG	895	-1.36	-5.69	1.24E-08	7.51E-07
IGHV3-73	145	-2.50	-5.68	1.37E-08	8.23E-07
IGKV1-27	228	-2.70	-5.67	1.39E-08	8.28E-07
IGKJ4	151	-2.94	-5.67	1.40E-08	8.29E-07
GVINP1	471	-1.20	-5.66	1.49E-08	8.77E-07
IGHV1-3	153	-2.63	-5.65	1.60E-08	9.36E-07
IGLL1	306	-2.06	-5.65	1.59E-08	9.36E-07
IGLJ3	102	-2.58	-5.63	1.80E-08	1.04E-06
ITGAL	1102	-1.06	-5.63	1.81E-08	1.05E-06
IGKC	23344	-2.68	-5.61	2.01E-08	1.15E-06
GPR183	234	-1.29	-5.60	2.11E-08	1.20E-06

IGKV2-28	772	-2.72	-5.59	2.26E-08	1.28E-06
GGTA1	120	-1.36	-5.59	2.27E-08	1.28E-06
IGKV1-39	1287	-3.04	-5.59	2.30E-08	1.30E-06
IGKV2D-28	774	-2.71	-5.58	2.44E-08	1.37E-06
PLPPR4	289	-1.77	-5.58	2.45E-08	1.37E-06
IGKV1-12	672	-2.91	-5.56	2.63E-08	1.45E-06
ITK	315	-1.41	-5.56	2.63E-08	1.45E-06
TNFSF13B	182	-1.52	-5.56	2.68E-08	1.47E-06
MXRA5	1567	-1.59	-5.56	2.72E-08	1.48E-06
IGKV3-11	1105	-2.67	-5.53	3.26E-08	1.75E-06
IGLV1-36	211	-2.68	-5.52	3.41E-08	1.83E-06
FAM30A	135	-2.14	-5.51	3.59E-08	1.92E-06
IGKV1D-16	506	-2.97	-5.51	3.68E-08	1.97E-06
NREP	268	-1.33	-5.48	4.16E-08	2.19E-06
IGHV1-24	187	-2.82	-5.47	4.53E-08	2.34E-06
SLC8A1	473	-1.28	-5.46	4.74E-08	2.44E-06
CD84	373	-1.27	-5.45	4.94E-08	2.54E-06
CD48	435	-1.40	-5.45	5.14E-08	2.63E-06
IGKV1D-12	661	-2.87	-5.44	5.27E-08	2.69E-06
CD52	634	-1.34	-5.42	5.83E-08	2.94E-06
IGKV2D-30	282	-2.86	-5.42	6.06E-08	3.03E-06
MAP4K1	301	-1.07	-5.42	6.12E-08	3.05E-06

GPR65	130	-1.35	-5.41	6.20E-08	3.09E-06
IGKV3D-11	896	-2.69	-5.41	6.45E-08	3.19E-06
SFRP4	611	-3.05	-5.40	6.58E-08	3.23E-06
IGKV3D-20	798	-2.78	-5.40	6.63E-08	3.24E-06
FGL2	1696	-1.31	-5.40	6.70E-08	3.26E-06
ARHGAP15	232	-1.28	-5.39	7.18E-08	3.47E-06
WDFY4	396	-1.37	-5.38	7.35E-08	3.54E-06
TMC8	733	-1.25	-5.37	7.73E-08	3.71E-06
IGHV3-23	906	-2.77	-5.34	9.44E-08	4.45E-06
IGLV1-44	731	-2.62	-5.33	9.55E-08	4.48E-06
FCRL5	100	-2.24	-5.33	9.78E-08	4.56E-06
IGLV3-1	461	-2.41	-5.32	1.03E-07	4.76E-06
LTB	185	-1.86	-5.32	1.04E-07	4.83E-06
IGHV3-15	305	-2.71	-5.30	1.14E-07	5.27E-06
IGKV2-24	164	-2.75	-5.30	1.17E-07	5.36E-06
ITIH5	850	-1.89	-5.29	1.20E-07	5.50E-06
MUC13	2187	-1.32	-5.29	1.21E-07	5.52E-06
IGKV3-20	1887	-2.72	-5.29	1.22E-07	5.55E-06
TPSAB1	1466	-1.48	-5.28	1.27E-07	5.69E-06
IGKV2-30	344	-2.83	-5.27	1.33E-07	5.96E-06
MZB1	299	-1.71	-5.27	1.34E-07	5.98E-06
DIO2	843	-2.15	-5.27	1.34E-07	5.99E-06

IGHV3-74	299	-2.53	-5.24	1.63E-07	7.21E-06
COL6A6	192	-1.72	-5.24	1.65E-07	7.23E-06
IGHV4-61	624	-2.44	-5.23	1.70E-07	7.47E-06
VASH1	655	-1.08	-5.21	1.87E-07	8.07E-06
IGHV4-34	248	-2.45	-5.20	1.94E-07	8.31E-06
NA	289	-1.36	-5.20	2.00E-07	8.55E-06
PDE4B	265	-1.30	-5.19	2.11E-07	9.00E-06
GZMH	134	-1.44	-5.19	2.14E-07	9.07E-06
IGHV3-49	224	-2.73	-5.18	2.18E-07	9.22E-06
TPSB2	1643	-1.47	-5.18	2.21E-07	9.33E-06
PARP15	244	-1.11	-5.18	2.22E-07	9.33E-06
CD247	226	-1.24	-5.17	2.32E-07	9.61E-06
IGHV4-39	523	-2.51	-5.17	2.36E-07	9.74E-06
IGHV3-33	980	-2.65	-5.17	2.38E-07	9.81E-06
DOCK2	679	-1.24	-5.16	2.45E-07	1.01E-05
TBC1D10C	441	-1.09	-5.16	2.50E-07	1.03E-05
IGLV1-47	660	-2.50	-5.15	2.56E-07	1.05E-05
ZBP1	101	-1.51	-5.15	2.60E-07	1.06E-05
ROBO2	365	-1.92	-5.15	2.63E-07	1.07E-05
GBP5	1043	-1.17	-5.14	2.71E-07	1.10E-05
GZMA	253	-1.21	-5.13	2.89E-07	1.17E-05
IGHV5-51	316	-2.56	-5.13	2.93E-07	1.18E-05

IL32	775	-1.55	-5.12	3.01E-07	1.21E-05
IGLV2-18	357	-2.53	-5.11	3.23E-07	1.28E-05
CTSW	175	-1.24	-5.11	3.27E-07	1.29E-05
ABI3BP	1371	-1.57	-5.11	3.30E-07	1.30E-05
PLEK	620	-1.18	-5.10	3.45E-07	1.35E-05
CXCL9	1772	-2.90	-5.09	3.54E-07	1.37E-05
IRF4	217	-1.78	-5.08	3.85E-07	1.48E-05
ADAM19	400	-1.61	-5.07	3.90E-07	1.49E-05
IGHV3-20	192	-2.67	-5.07	3.94E-07	1.50E-05
IGHV3-13	179	-2.51	-5.07	4.00E-07	1.52E-05
FYB1	777	-1.29	-5.06	4.21E-07	1.59E-05
MAF	331	-1.01	-5.03	4.91E-07	1.82E-05
IGKV1-33	744	-2.82	-5.02	5.11E-07	1.88E-05
IGKV1D-33	746	-2.81	-5.01	5.53E-07	2.01E-05
CD28	114	-1.90	-5.00	5.66E-07	2.06E-05
IGKV1D-13	393	-2.65	-5.00	5.72E-07	2.08E-05
CD22	154	-1.64	-4.98	6.39E-07	2.31E-05
MCOLN2	210	-1.08	-4.97	6.73E-07	2.41E-05
SEPTIN1	337	-1.09	-4.97	6.73E-07	2.41E-05
TGFB3	627	-1.00	-4.97	6.80E-07	2.43E-05
IGKV1-5	1078	-2.60	-4.97	6.83E-07	2.43E-05
JAML	795	-1.13	-4.96	6.91E-07	2.46E-05

TNFSF14	189	-1.11	-4.96	7.06E-07	2.50E-05
IGLV2-34	320	-2.26	-4.94	7.85E-07	2.75E-05
IGHV3-7	662	-2.70	-4.92	8.44E-07	2.92E-05
DCHS2	155	-1.36	-4.92	8.44E-07	2.92E-05
LAIR1	382	-1.07	-4.92	8.67E-07	2.99E-05
HLA-DOA	1286	-1.03	-4.92	8.66E-07	2.99E-05
IKZF1	697	-1.10	-4.92	8.86E-07	3.05E-05
LCP1	3622	-1.18	-4.90	9.49E-07	3.23E-05
UBD	1644	-1.66	-4.90	9.71E-07	3.30E-05
COL14A1	1515	-1.60	-4.88	1.03E-06	3.50E-05
PARVG	535	-1.14	-4.88	1.04E-06	3.52E-05
HLA-DOB	121	-1.40	-4.86	1.20E-06	3.97E-05
MS4A6A	704	-1.07	-4.85	1.22E-06	4.01E-05
IGHV1-2	339	-2.66	-4.85	1.21E-06	4.01E-05
CARMIL2	163	-1.29	-4.84	1.33E-06	4.35E-05
HSPA7	295	-1.18	-4.83	1.34E-06	4.37E-05
OASL	178	-1.40	-4.83	1.37E-06	4.46E-05
IGHV4-28	141	-2.63	-4.83	1.37E-06	4.48E-05
TRAF3IP3	415	-1.08	-4.82	1.42E-06	4.60E-05
GNG2	328	-1.20	-4.82	1.46E-06	4.71E-05
IGHV4-59	655	-2.38	-4.81	1.53E-06	4.89E-05
DOK2	182	-1.12	-4.80	1.57E-06	5.02E-05

IGHV3-30	938	-2.53	-4.77	1.80E-06	5.66E-05
IGLV2-23	1054	-2.19	-4.75	2.05E-06	6.32E-05
HMCN1	1660	-1.38	-4.73	2.28E-06	6.97E-05
AGAP2	141	-1.19	-4.73	2.28E-06	6.97E-05
P2RY13	209	-1.33	-4.70	2.54E-06	7.63E-05
IGLC7	666	-2.35	-4.69	2.68E-06	8.02E-05
IGHV2-5	149	-2.67	-4.68	2.83E-06	8.42E-05
IFI44L	1914	-1.28	-4.67	2.95E-06	8.73E-05
TPSD1	222	-1.39	-4.67	3.00E-06	8.84E-05
MMP9	101	-2.15	-4.67	3.02E-06	8.87E-05
IGLC3	8964	-2.26	-4.66	3.17E-06	9.24E-05
IGLV3-9	121	-2.63	-4.65	3.25E-06	9.41E-05
CORO1A	1616	-1.12	-4.64	3.48E-06	9.97E-05
CD7	197	-1.33	-4.62	3.75E-06	0.0001
CCL19	838	-2.77	-4.58	4.59E-06	0.0001
FGF1	139	-1.50	-4.58	4.69E-06	0.0001
LAMA4	1644	-1.26	-4.57	4.78E-06	0.0001
COL10A1	203	-1.26	-4.57	4.91E-06	0.0001
EXOC3L4	157	-1.02	-4.57	4.99E-06	0.0001
OLFML2B	324	-1.19	-4.52	6.05E-06	0.0002
IGLV2-11	811	-2.36	-4.52	6.06E-06	0.0002
GBP4	2156	-1.01	-4.51	6.58E-06	0.0002

SLITRK5	116	-1.19	-4.50	6.83E-06	0.0002
RAC2	950	-1.06	-4.49	7.00E-06	0.0002
TFEC	124	-1.12	-4.45	8.76E-06	0.0002
IGLV7-46	101	-2.68	-4.44	9.04E-06	0.0002
PSTPIP1	195	-1.10	-4.43	9.36E-06	0.0002
IGLV2-14	1781	-2.27	-4.43	9.49E-06	0.0002
IGLL5	1198	-2.26	-4.41	1.03E-05	0.0003
IGHA2	7043	-2.08	-4.40	1.10E-05	0.0003
BTK	207	-1.15	-4.40	1.10E-05	0.0003
IGHA1	21057	-1.97	-4.39	1.13E-05	0.0003
COL1A2	4278	-2.00	-4.37	1.24E-05	0.0003
MS4A1	361	-1.48	-4.36	1.30E-05	0.0003
IGLV2-8	858	-2.32	-4.35	1.34E-05	0.0003
IGLV6-57	182	-2.41	-4.34	1.41E-05	0.0003
VCAM1	351	-2.08	-4.34	1.42E-05	0.0003
GUCY1A1	779	-1.28	-4.34	1.43E-05	0.0003
ECM2	493	-1.11	-4.31	1.60E-05	0.0004
MRC2	872	-1.26	-4.31	1.67E-05	0.0004
AIF1	471	-1.09	-4.30	1.71E-05	0.0004
GUCY1A2	178	-1.80	-4.27	1.98E-05	0.0004
IGLV3-19	292	-2.58	-4.26	2.03E-05	0.0005
IGLC2	11569	-2.16	-4.25	2.15E-05	0.0005

LINC00861	116	-1.33	-4.25	2.17E-05	0.0005
ITLN1	171	-1.70	-4.24	2.28E-05	0.0005
CYBB	986	-1.16	-4.23	2.35E-05	0.0005
MYO1G	305	-1.08	-4.23	2.36E-05	0.0005
HCST	131	-1.08	-4.22	2.42E-05	0.0005
IGLV3-25	461	-2.37	-4.21	2.59E-05	0.0006
CXCL14	1612	-1.69	-4.20	2.67E-05	0.0006
ST8SIA6	120	-1.01	-4.20	2.72E-05	0.0006
CALHM6	166	-1.11	-4.19	2.85E-05	0.0006
TNFAIP8L2	112	-1.00	-4.18	2.93E-05	0.0006
SLCO2B1	822	-1.02	-4.17	2.99E-05	0.0006
GAPT	104	-1.08	-4.17	3.00E-05	0.0006
IGLV3-21	537	-2.08	-4.15	3.35E-05	0.0007
POSTN	3332	-1.07	-4.14	3.45E-05	0.0007
COL3A1	4570	-2.19	-4.14	3.50E-05	0.0007
LUM	1511	-2.45	-4.13	3.56E-05	0.0007
COL1A1	2141	-1.21	-4.13	3.62E-05	0.0007
LILRB1	247	-1.17	-4.13	3.65E-05	0.0007
FREM1	220	-1.59	-4.07	4.66E-05	0.0009
PTGDS	1569	-2.07	-4.06	4.83E-05	0.001
CXCL10	1155	-2.14	-4.06	4.92E-05	0.001
NTRK2	298	-1.13	-3.99	6.56E-05	0.001

CCL21	326	-2.04	-3.98	7.03E-05	0.001
SIGLEC1	217	-1.11	-3.97	7.21E-05	0.001
P2RX7	112	-1.00	-3.96	7.39E-05	0.001
EDNRA	635	-1.04	-3.91	9.37E-05	0.002
HGF	151	-1.61	-3.90	9.68E-05	0.002
IGHD	1230	-1.83	-3.87	0.0001	0.002
ENPP2	666	-1.24	-3.85	0.0001	0.002
F2RL2	166	-1.28	-3.79	0.0001	0.002
SLC2A14	123	-1.13	-3.79	0.0002	0.002
NKG7	230	-1.22	-3.78	0.0002	0.003
CXCL12	1272	-1.58	-3.77	0.0002	0.003
PTGS1	437	-1.05	-3.77	0.0002	0.003
CTHRC1	101	-1.38	-3.73	0.0002	0.003
CST7	123	-1.09	-3.69	0.0002	0.003
PRRX1	757	-1.26	-3.66	0.0003	0.004
MATN2	1086	-1.02	-3.66	0.0003	0.004
F13A1	1017	-1.65	-3.66	0.0003	0.004
CD79A	220	-1.56	-3.64	0.0003	0.004
ASPN	719	-1.63	-3.62	0.0003	0.004
LSAMP	674	-1.32	-3.61	0.0003	0.004
LST1	257	-1.01	-3.54	0.0004	0.005
TMEM119	351	-1.52	-3.51	0.0004	0.006

TFF1	113	-1.30	-3.51	0.0004	0.006
CSF2RB	701	-1.18	-3.51	0.0005	0.006
COL5A1	923	-1.42	-3.50	0.0005	0.006
OLFML1	306	-1.13	-3.50	0.0005	0.006
CHI3L2	187	-1.52	-3.50	0.0005	0.006
LDB2	637	-1.11	-3.45	0.0006	0.007
JAK3	641	-1.01	-3.45	0.0006	0.007
SGIP1	107	-1.50	-3.44	0.0006	0.007
CXCL11	313	-2.05	-3.43	0.0006	0.008
CPNE5	140	-1.01	-3.41	0.0007	0.008
ADGRA2	810	-1.35	-3.40	0.0007	0.008
FNDC1	113	-1.39	-3.39	0.0007	0.008
IGHM	2170	-1.54	-3.38	0.0007	0.009
EGFLAM	116	-1.27	-3.36	0.0008	0.009
PLA2G4C	205	-1.09	-3.35	0.0008	0.010
CEACAM5	2470	-1.07	-3.33	0.0009	0.010
JAM2	720	-1.10	-3.28	0.0010	0.012
CLIC2	381	-1.00	-3.27	0.0011	0.012
PI16	645	-1.54	-3.23	0.0012	0.013
FAM111B	100	-1.16	-3.21	0.0013	0.014
ABCB1	521	-1.08	-3.19	0.0014	0.015
MMP10	3182	-1.01	-3.19	0.0014	0.015

DPT	1681	-1.72	-3.18	0.0014	0.015
TSPAN18	337	-1.04	-3.18	0.0015	0.015
COL15A1	2580	-1.70	-3.17	0.0015	0.016
OGN	554	-1.47	-3.15	0.0016	0.016
EPHA3	338	-1.13	-3.15	0.0016	0.016
FRZB	237	-1.27	-3.12	0.0018	0.018
CD248	132	-1.18	-3.08	0.0020	0.019
GLI1	116	-1.10	-3.06	0.0022	0.021
COL8A1	243	-1.20	-3.05	0.0023	0.021
PREX2	323	-1.35	-3.01	0.0026	0.024
CACNA1C	281	-1.04	-2.99	0.0028	0.025
BGN	2401	-1.49	-2.98	0.0028	0.025
COLEC12	198	-1.02	-2.98	0.0029	0.025
SPON1	1268	-1.14	-2.98	0.0029	0.025
CLEC3B	216	-1.17	-2.98	0.0029	0.025
SELE	306	-1.67	-2.97	0.0029	0.026
TEK	353	-1.15	-2.92	0.0035	0.029
FAT4	1574	-1.15	-2.86	0.0042	0.034
FLRT2	373	-1.46	-2.85	0.0043	0.035
IDO1	1607	-1.46	-2.85	0.0044	0.035
HAS2	115	-1.18	-2.83	0.0047	0.037
BCHE	209	-1.68	-2.82	0.0048	0.038

MEOX1	415	-1.45	-2.81	0.0049	0.038
DNM3OS	178	-1.04	-2.81	0.0049	0.038
ZNF385D	246	-1.30	-2.81	0.0050	0.038
COL6A2	3477	-1.39	-2.79	0.0053	0.040
SELP	623	-1.22	-2.76	0.0057	0.043
GREM2	293	-1.12	-2.76	0.0058	0.043
HPSE2	200	-1.43	-2.74	0.0062	0.045
MOXD1	438	-1.21	-2.73	0.0064	0.046

Supplementary Table E5 Geneset enrichment analysis for top 10 differentially enriched gene ontology pathways in bronchial brushes

Gene ontology (GO) pathways significantly differentially expressed after 4 weeks of inhaled fluticasone (treatment group only) using Limma. Pathways shown are censored at Benjamini Hochberg adjusted $P < 0.05$ and ordered by P value. Under figure *x* axis represents approximate gene ranks. NES, normalised enrichment score.

Brushings	GO Pathway	Gene ranks	NES	p	p adj
Upregulated by ICS	Positive regulation of ERBB signaling pathway		1.93	0.0004276	0.0450544
	Cytokine activity		1.59	0.00064	0.053437
	Positive regulation of T cell mediated cytotoxicity		2.02	0.0008298	0.0626609
	Regulation of B cell receptor signaling pathway		1.96	0.0019826	0.1082716
	Cytokine mediated signaling pathway		1.39	0.0021439	0.1101276
	Positive regulation of adaptive immune response		1.60	0.0028807	0.1314115
	Regulation of T cell mediated cytotoxicity		1.86	0.0031884	0.1383193
	Tumor necrosis factor mediated signaling pathway		1.56	0.0043835	0.1731496
	Regulation of interferon gamma production		1.56	0.0047389	0.1766441
	Mannosidase activity		1.87	0.0048731	0.1782097
Downregulated by ICS	Adaptive immune response		-1.99	4.291E-09	1.502E-05
	Lymphocyte activation		-1.91	6.788E-09	1.502E-05
	Regulation of immune effector process		-1.81	1.274E-08	1.879E-05
	Immune effector process		-1.81	2.633E-08	2.913E-05
	Leukocyte activation		-1.78	1.474E-07	0.0001304
	Lymphocyte mediated immunity		-2.04	1.793E-07	0.0001322
	Leukocyte mediated immunity		-1.94	4.771E-07	0.0003016
	Sodium ion homoeostasis		-2.29	1.517E-06	0.0008388
	Activation of immune response		-1.67	2.8E-06	0.0013549
	Positive regulation of cell activation		-1.74	3.062E-06	0.0013549

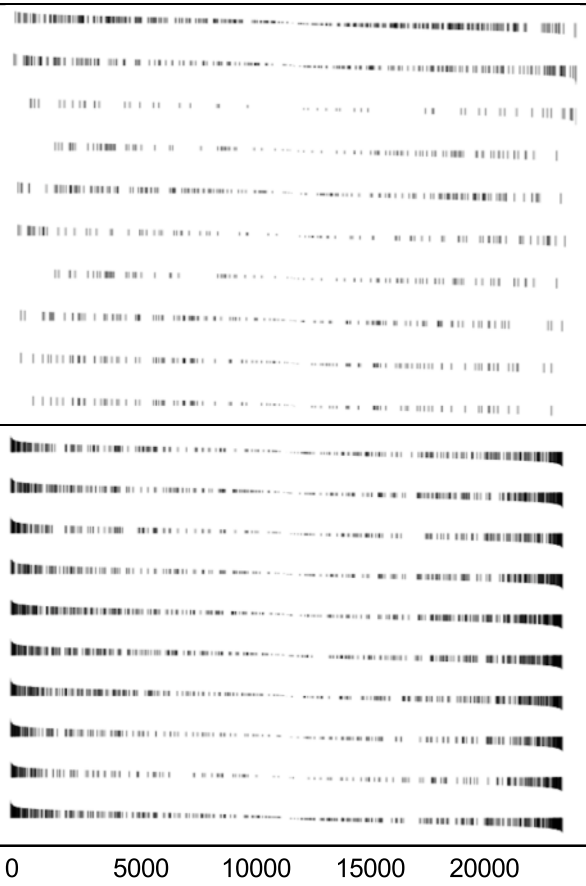

Supplementary Table E6. Geneset enrichment analysis for top 10 differentially enriched gene ontology pathways in bronchial biopsies.

Gene ontology (GO) pathways significantly differentially expressed after 4 weeks of inhaled fluticasone (treatment group only) using Limma. Pathways shown are censored at Benjamini Hochberg adjusted $P < 0.05$ and ordered by P value. Under figure x axis represents approximate gene ranks. NES, normalised enrichment score.

Biopsies	GO Pathway	Gene ranks	NES	p	p adj
Upregulated by ICS	Mitochondrial matrix		2.03	1.90711E-12	6.71695E-11
	Oxidoreductase activity acting on the CH CH group of donors		2.50	5.46935E-09	1.1432E-07
	Cofactor binding		1.98	1.12177E-08	2.1816E-07
	Mitochondrial translation		2.31	1.53754E-08	2.88968E-07
	Cilium organization		2.08	2.39225E-08	4.2974E-07
	Organellar ribosome		2.31	3.52179E-08	6.03562E-07
	DNA Packaging complex		2.25	6.07677E-08	9.95656E-07
	Cellular respiration		2.12	1.22605E-07	1.83199E-06
	Translational termination		2.25	1.6989E-07	2.41244E-06
	Mitochondrial protein complex		2.12	3.30354E-07	4.42417E-06
Downregulated by ICS	Regulation of cell activation		-3.28	3.83652E-67	1.71607E-63
	Adaptive immune response		-3.45	3.00654E-57	5.21315E-54
	Positive regulation of cell activation		-3.38	3.49641E-57	5.21315E-54
	Side of membrane		-3.16	1.28859E-54	1.44097E-51
	Activation of immune response		-3.14	2.03234E-54	1.81814E-51
	Immune effector process		-3.07	3.35375E-53	2.50022E-50
	Regulation of homotypic cell cell adhesion		-3.24	2.82053E-50	1.80232E-47
	Lymphocyte activation		-3.19	9.17767E-49	5.13147E-46
	Leukocyte activation		-3.09	1.07142E-48	5.32495E-46
	Regulation of cell cell adhesion		-3.12	2.97918E-47	1.33259E-44

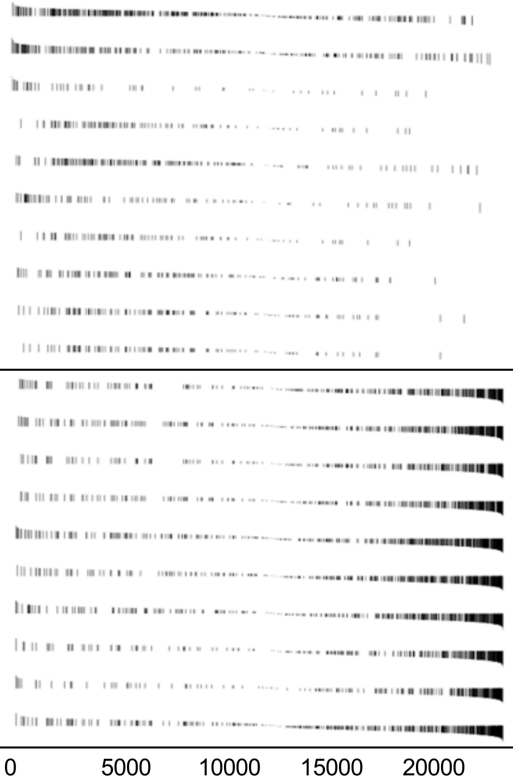

Supplementary Table E7. Geneset enrichment analysis for top 6 differentially enriched type 2 immunity related genes in bronchial biopsies stratified by atopic status.

Type 2 immunity related genes from the top 20 most downregulated genes in bronchial biopsies, stratified by atopic status, after 4 weeks of inhaled fluticasone (treatment group only). Log2 fold changes and statistical tests calculated using Wald tests in *DESeq2*. Genes shown are censored at FDR P < 0.05 and log(2) fold change of ±1 and ordered by P value. FDR, false discovery rate.

	Atopic						
**Gene**	**Baseline read count**	**Log2 fold change**	**t**	***P* value**	**FDR *P* value**	**Comments and aliases**	**Functional role**
FCER1A	222	-2.73	-6.73E+00	1.66865E-11	7E-09	High affinity IgE receptor; expressed on airway mast cells and dendritic cells.	Type 2 immunity
CLEC10A	140	-2.71	-7.49E+00	7.12098E-14	5.53E-11	C-Type Lectin Domain Containing 10A; CD301: marker for alternatively activated macrophages.	Type 2 immunity
SERPINB10	461	-1.98	-5.95E+00	2.71106E-09	5.81E-07	Serpin Family B Member 10; inhibits Th2 cell apoptosis, highly upregulated on airway epithelium by IL-13.	Type 2 immunity
CCR5	249	-2.24	-4.81E+00	1.54656E-06	0.000135	Ligands include CCL5 (RANTES), MCP-2, MIP-1a, MIP-1b. Chemotactic for T2–induced eosinophils.	Type 2 immunity
CPA3	1183	-2.44	-5.95E+00	2.67853E-09	5.81E-07	Carboxypeptidase A3: mast cell-specific peptidase located in secretory granules.	Type 2 immunity
IL33	5828	-1.27	-1.11E+01	1.44025E-28	1.34E-24	Interleukin 33; airway epithelial cell alarmin which acts via ST2 to promote type-2 responses.	Type 2 immunity
	Non-atopic						
**Gene**	**Baseline read count**	**Log2 fold change**	**t**	***P* value**	**FDR *P* value**	**Comments and aliases**	**Functional role**
FCER1A	229	-2.09	-1.05E+01	1.11539E-25	5.26E-22	High affinity IgE receptor; expressed on airway mast cells and dendritic cells.	Type 2 immunity
CLEC10A	220	-2.01	-6.30E+00	2.91008E-10	8.57E-08	C-Type Lectin Domain Containing 10A; CD301: marker for alternatively activated macrophages.	Type 2 immunity
SERPINB10	303	-1.24	-6.38E+00	1.7865E-10	5.71E-08	Serpin Family B Member 10; inhibits Th2 cell apoptosis, highly upregulated on airway epithelium by IL-13.	Type 2 immunity
CCR5	494	-1.80	-6.68E+00	2.4352E-11	9.56E-09	Ligands include CCL5 (RANTES), MCP-2, MIP-1a, MIP-1b. Chemotactic for T2–induced eosinophils.	Type 2 immunity
CPA3	796	-1.81	-5.33E+00	1.00291E-07	1.07E-05	Carboxypeptidase A3: mast cell-specific peptidase located in secretory granules.	Type 2 immunity
IL33	7389	-1.22	-6.63E+00	3.36904E-11	1.27E-08	Interleukin 33; airway epithelial cell alarmin which acts via ST2 to promote type-2 responses.	Type 2 immunity

Supplementary Table E8. Cell deconvolution analysis.

Cell deconvolution analysis was performed on bronchial biopsies using MuSiC to predict changes in cell numbers between day 0 and week 4 of ICS therapy, ordered by geneset normalised enrichment score.

Biopsies	Geneset	Gene ranks	NES	p	p adj
Upregulated by ICS	Lipofibroblast		1.25	0.085	0.1
	Differentiating basal cells		1.16	0.13	0.15
	Alveolar type 2 epithelial cells		1.15	0.17	0.2
	Neuroendocrine cells		0.98	0.5	0.6
	Proliferating NKT cells		0.94	0.6	0.7
	Fibromyocyte cells		0.87	0.7	0.7
	Lung serous cells		0.90	0.7	0.7
Downregulated by ICS	Plasmacytoid dendritic cells		-2.34	7.50E-10	3.20E-09
	Lung basophil/ mast cells		-2.04	4.80E-10	2.30E-09
	Lung CD8 effector memory T cells		-2.60	2.60E-10	1.40E-09
	Lung CD4 naïve T cells		-2.38	6.00E-11	3.60E-10
	Lung natural killer T cells		-2.72	3.50E-12	2.40E-11
	Lung classical monocytes		-2.30	1.80E-14	1.50E-13
	Lung natural killer cells		-2.91	1.80E-21	1.90E-20
	Lung B cells		-2.83	8.30E-22	1.20E-20
	Lung CD4 effector memory cells		-3.06	2.30E-22	4.80E-21
	Lung CD8 naïve T cells		-3.30	1.20E-35	4.90E-34

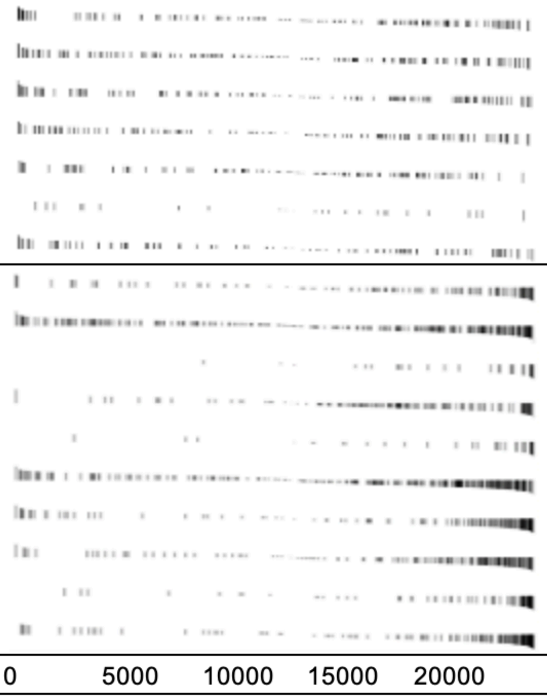

Supplementary Table E9. Estimated cell type-specific effects of ICS.

Cell-type specific differential gene expression effects of ICS were estimated using TOAST in R, on proportions of 5 different epithelial and structural cell types (multiciliated, basal resting, peribronchial fibroblasts, club and suprabasal), estimated using MuSiC deconvolution. Genes with FDR P<0.05 are shown, ranked by FDR. TOAST defines the effect size as $\beta/(\mu+\beta/2)$, where μ is base-line expression in one group and β is the gene expression difference between two group.

No genes passed FDR P <0.05 for club, peribronchial fibroblasts, basal resting cells in bronchial biopsies, nor for any of these cell types in bronchial brushings.

Sample type	Bronchial biopsies											
Cell type	Suprabasal						Multiciliated epithelial cells					
ICS effects	Gene	Beta	Mu	Effect Size	*P* value	FDR *P* value	Gene	Beta	Mu	Effect Size	*P* value	FDR *P* value
	PLA2G2D	-9545	9949.49	-1.84	4E-06	0.032	FYB2	3420	4766	0.53	1E-06	0.014
	CD19	-25642	32105	-1.33	6E-06	0.032	PKIB	2944	1768	0.91	1E-06	0.014
	TRBC2	-174113	149746	-2.78	6E-06	0.032	KPNA7	393	541	0.53	2E-06	0.016
	SPIB	-31910	33126.7	-1.86	7E-06	0.032						
	TBC1D27P	-7883	8849	-1.61	1E-05	0.032						
	ADGRG5	-17595	16308	-2.34	1E-05	0.032						
	GRM4	-7938	7942	-2.00	1E-05	0.032						
	VPREB3	-5030	5814	-1.52	2E-05	0.032						
	DTX1	-8072	8683	-1.74	2E-05	0.032						
	LGALS2	-29090	23715	-3.17	2E-05	0.032						
	CD3D	-62103	52710	-2.87	2E-05	0.032						
	TLR10	-25362	27161	-1.75	2E-05	0.032						
	GNGT2	-12364	10994	-2.57	2E-05	0.032						
	MSC	-9841	7157	-4.40	2E-05	0.032						
	TNFRSF9	-5818	5590	-2.17	3E-05	0.034						
	UNC5B-AS1	7423	6748	0.71	3E-05	0.036						
	PDCD1	-14784	13583	-2.39	4E-05	0.04						
	TIFAB	-5028	4302	-2.81	4E-05	0.04						
	SEMA7A	-23895	25372	-1.78	4E-05	0.04						
	CCR6	-29650	26848	-2.47	4E-05	0.04						
	ZC3H12D	-13755	14627	-1.77	4E-05	0.042						
	NAPSB	-74057	66911	-2.48	5E-05	0.042						
	CTLA4	-13025	11011	-2.90	5E-05	0.044						
	CR2	-29721	34188	-1.54	5E-05	0.044						
	CXCR6	-67960	56639	-3.00	6E-05	0.045						
	TNFRSF13C	-3999	4804	-1.43	6E-05	0.045						
	FCRLA	-15398	20733	-1.18	6E-05	0.045						
	IL21R	-18561	15649	-2.91	6E-05	0.045						
	SNHG17	-25373	32515	-1.28	7E-05	0.045						
	PTPRCAP	-70464	68604	-2.11	7E-05	0.045						
	GPR55	-4601	4439	-2.15	7E-05	0.045						
	CXCR5	-12565	14297	-1.57	8E-05	0.048						
	LTB	-55931	50686	-2.46	8E-05	0.05						

Supplementary Figure E1

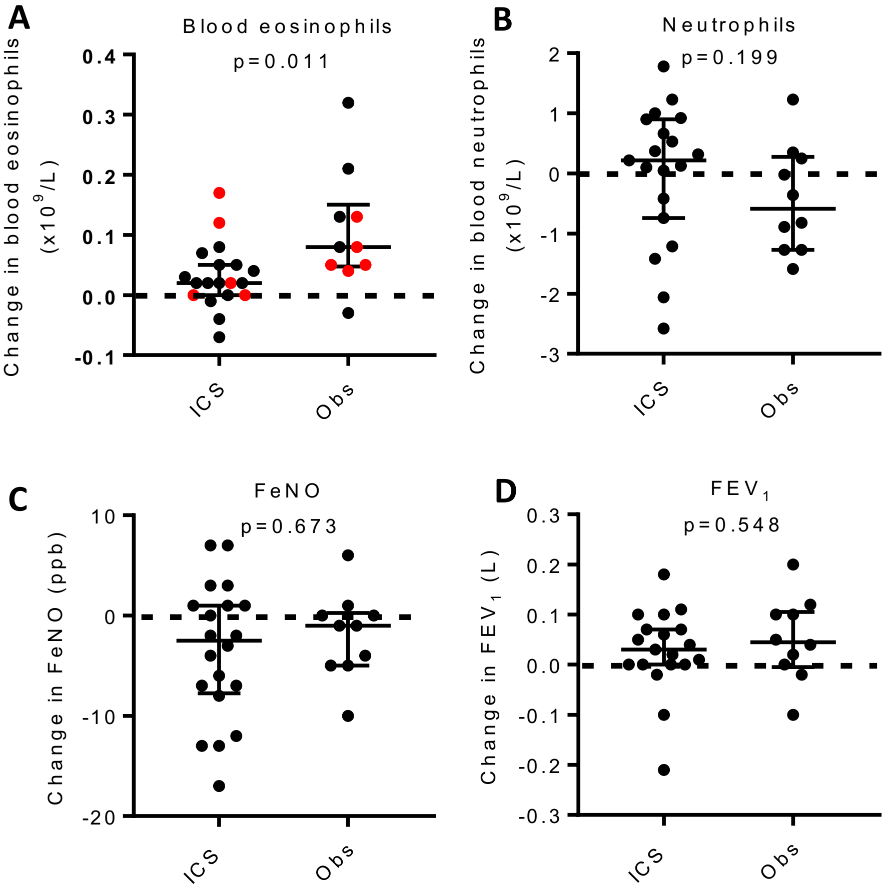

Supplementary Figure E1

The change in peripheral blood biomarkers and airway physiology on participants with available paired data before and after 4 weeks' treatment with inhaled fluticasone or without treatment. Changes in numbers of **A**) eosinophils, with atopic participants shown in red, or **B**) neutrophils in peripheral blood. Changes in **C**) fractional exhaled nitric oxide (FeNO) or **D**) forced expiratory volume in 1 second (FEV_1). Horizontal bars represent median (IQR), analysed by Mann Whitney U test. Obs represents the observation group.

Supplementary Figure E2

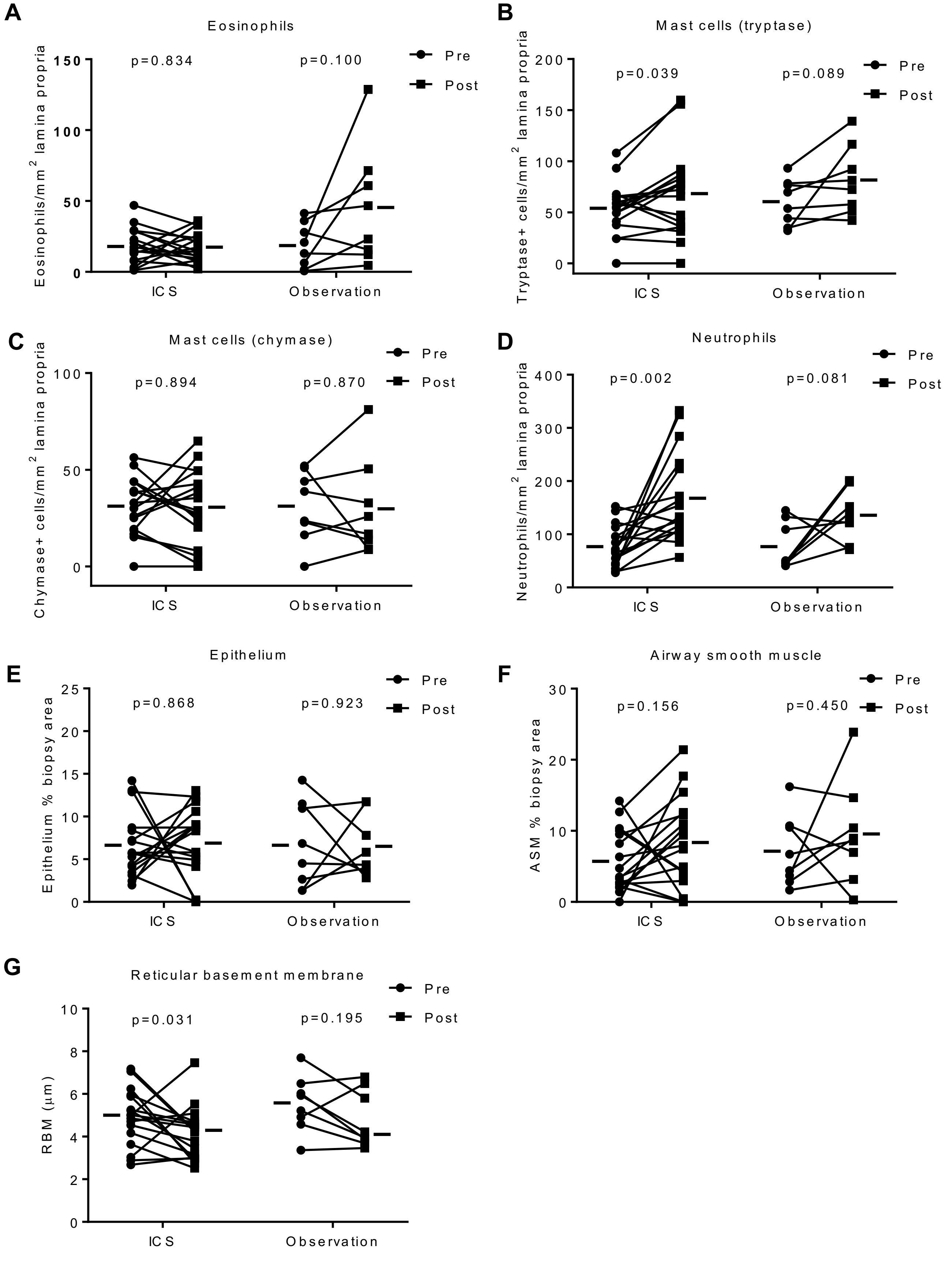

Supplementary Figure E2 (above)

Immunohistochemical analysis of the lamina propria showing cell counts and remodelling features from participants with available paired data before and after 4 weeks treatment with inhaled fluticasone or without treatment. Figure shows before and after data for numbers of **A**) eosinophils, with atopic participants shown in red, **B**) tryptase-positive mast cells, **C**) chymase-positive mast cells, or **D**) neutrophils, expressed in absolute counts/mm^2. Areas of **E**) epithelium or **F**) airway smooth muscle (ASM), expressed as a percentage of biopsy area. **G**) Measurements of reticular basement membrane (RBM) thickness. Horizontal bars represent mean (SD)(eosinophils, mast cells, neutrophils, ASM, epithelium) or median (IQR) (RBM), analysed by paired t test or Wilcoxon ranked pairs tests respectively.

Supplementary Figure E3

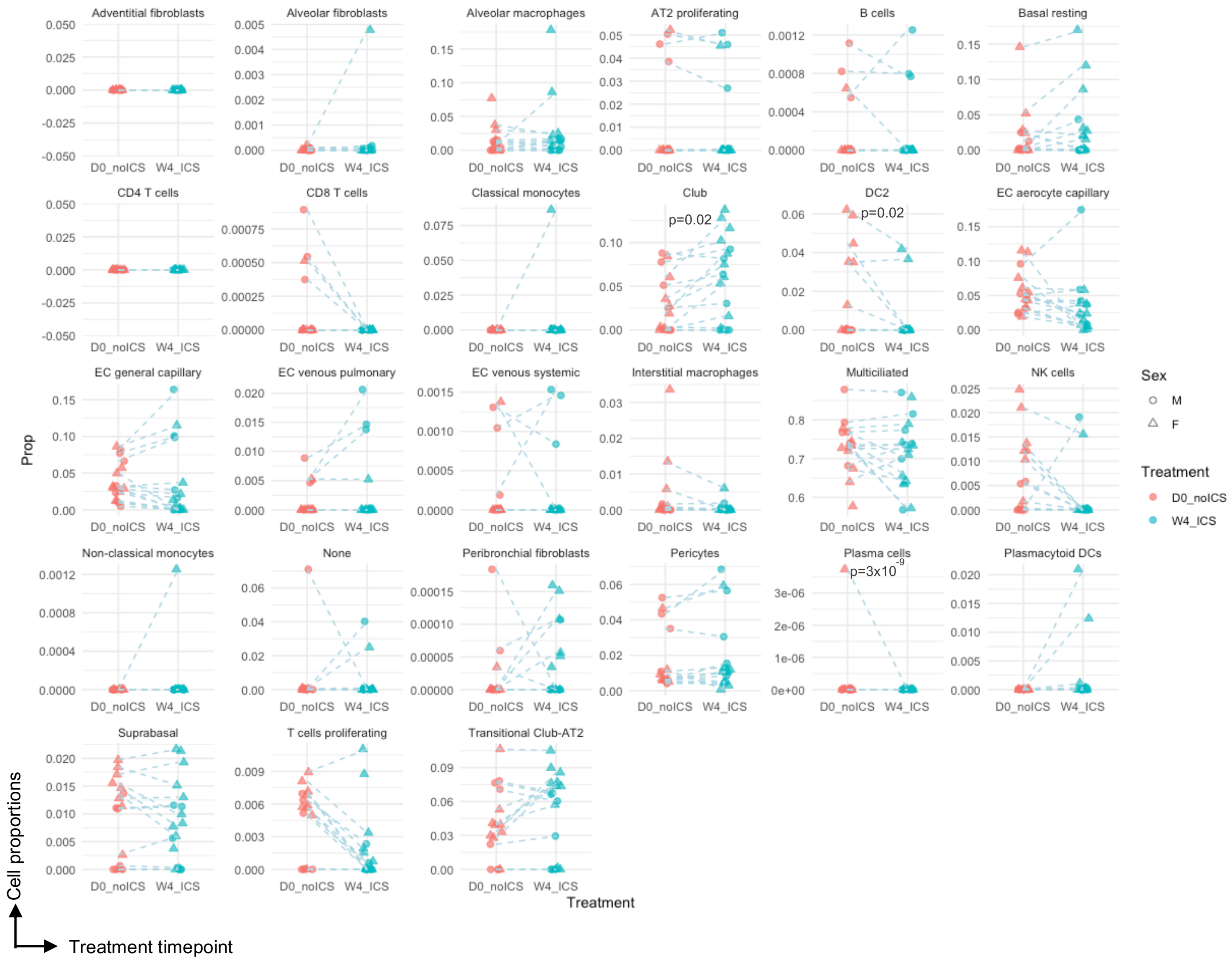

Supplementary Figure E3 (Brushes)

Cell deconvolution analysis of bronchial brushing transcriptomic data. Analysis performed using MuSiC and reference cell signatures from single cell data from the Lung Cell Atlas (E5) and annotated with Azimuth in. FDR P values, shown where P<0.05, represent paired non-parametric ranked tests from a linear regression analysis between week 4 and week 0.

Supplementary Figure E4

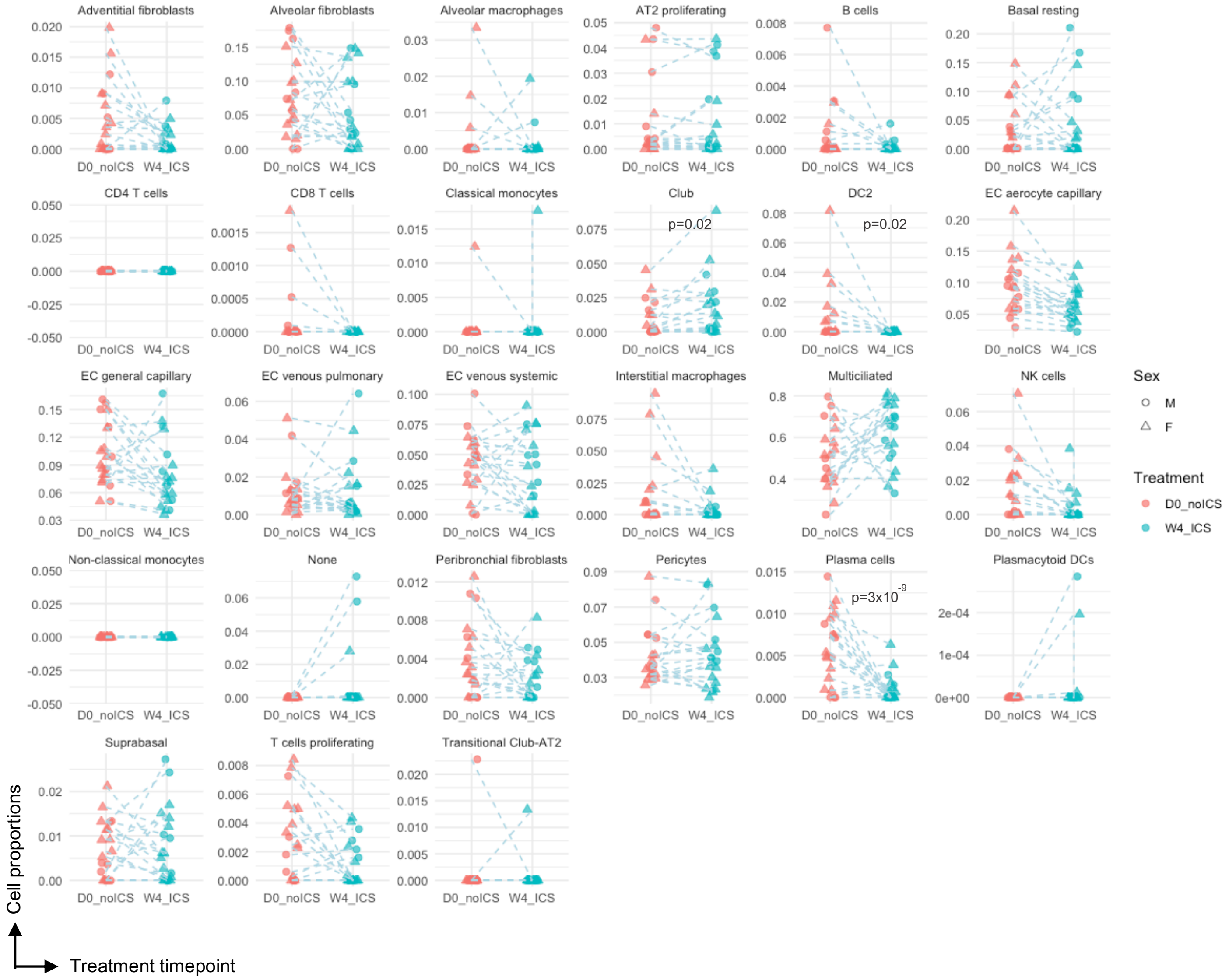

Supplementary Figure E4 (Biopsies)

Cell deconvolution analysis of bronchial biopsy transcriptomic data. Analysis performed using MuSiC and reference cell signatures from single cell data from the Lung Cell Atlas (E5) and annotated with Azimuth in. FDR P values, shown where P<0.05, represent paired non-parametric ranked tests from a linear regression analysis between week 4 and week 0.

Supplementary Figure E5

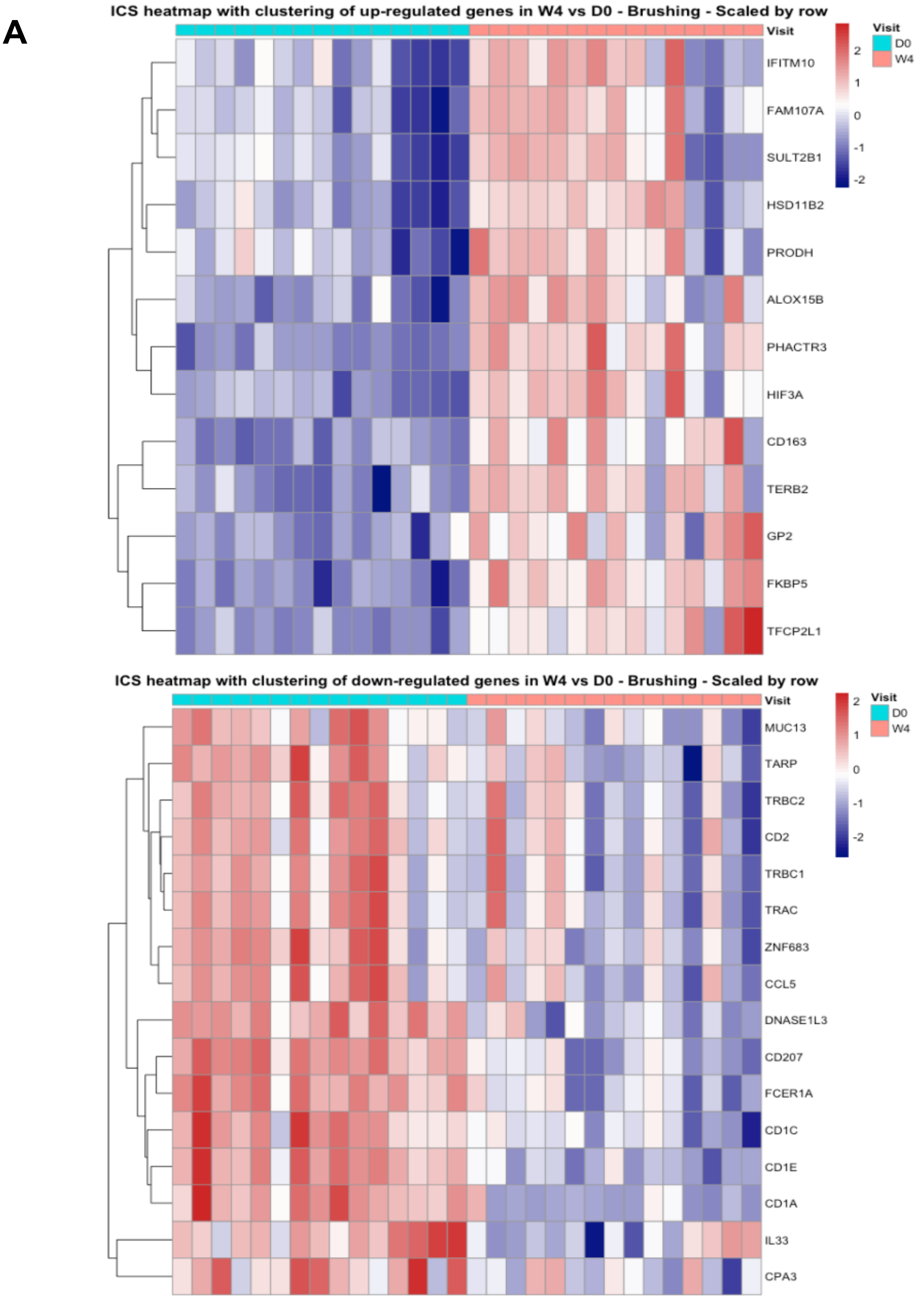

Supplementary Figure E5. Changes in gene expression measured by RNAseq in response to 4 weeks treatment with inhaled fluticasone. **A**) Bronchial brush heatmap representing the most differentially expressed genes (adjusted P<0.001, log2 fold change>0.5). **B**) Bronchial biopsy heatmap representing the most differentially expressed genes (adjusted P<0.001, log2 fold change>0.5).

Supplementary Figure E5 (continued)

B

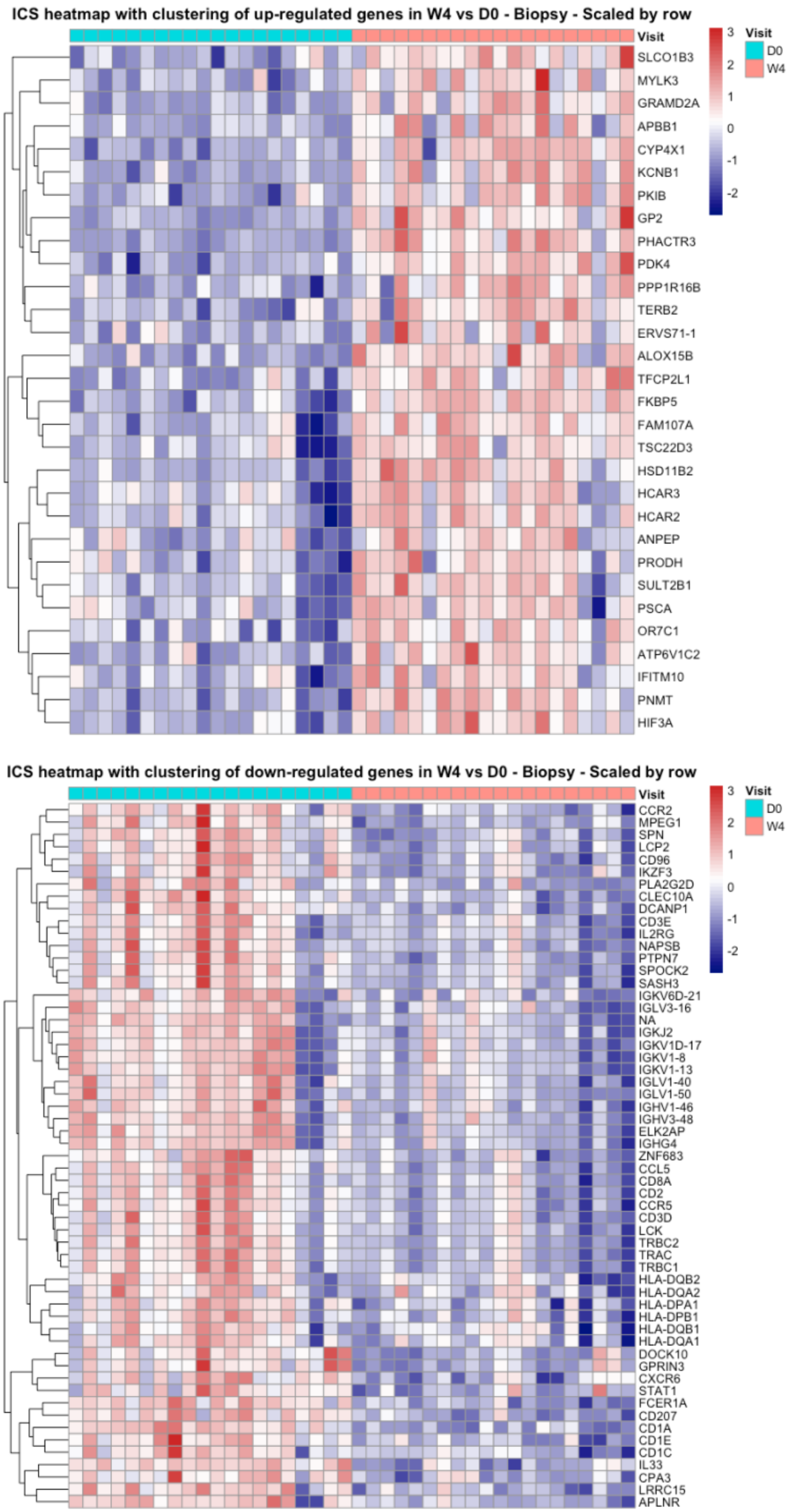

Supplementary Figure E6

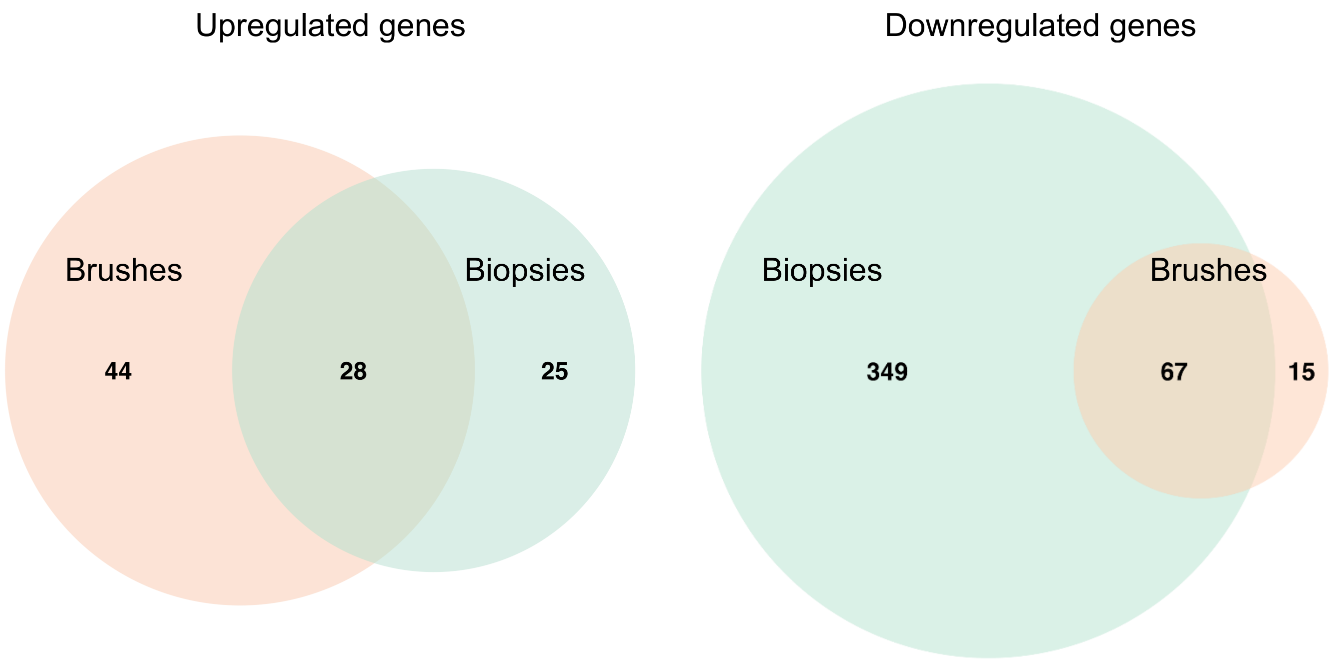

Supplementary Figure E6

Venn diagram of numbers of differentially expressed genes at week 4 versus baseline amongst those receiving inhaled fluticasone, showing genes **A**) upregulated and **B**) downregulated. Numbers generated with *DeSeq2*, with a significance threshold of log2 fold change > 1 and adjusted $p < 0.05$.

Supplementary Figure E7

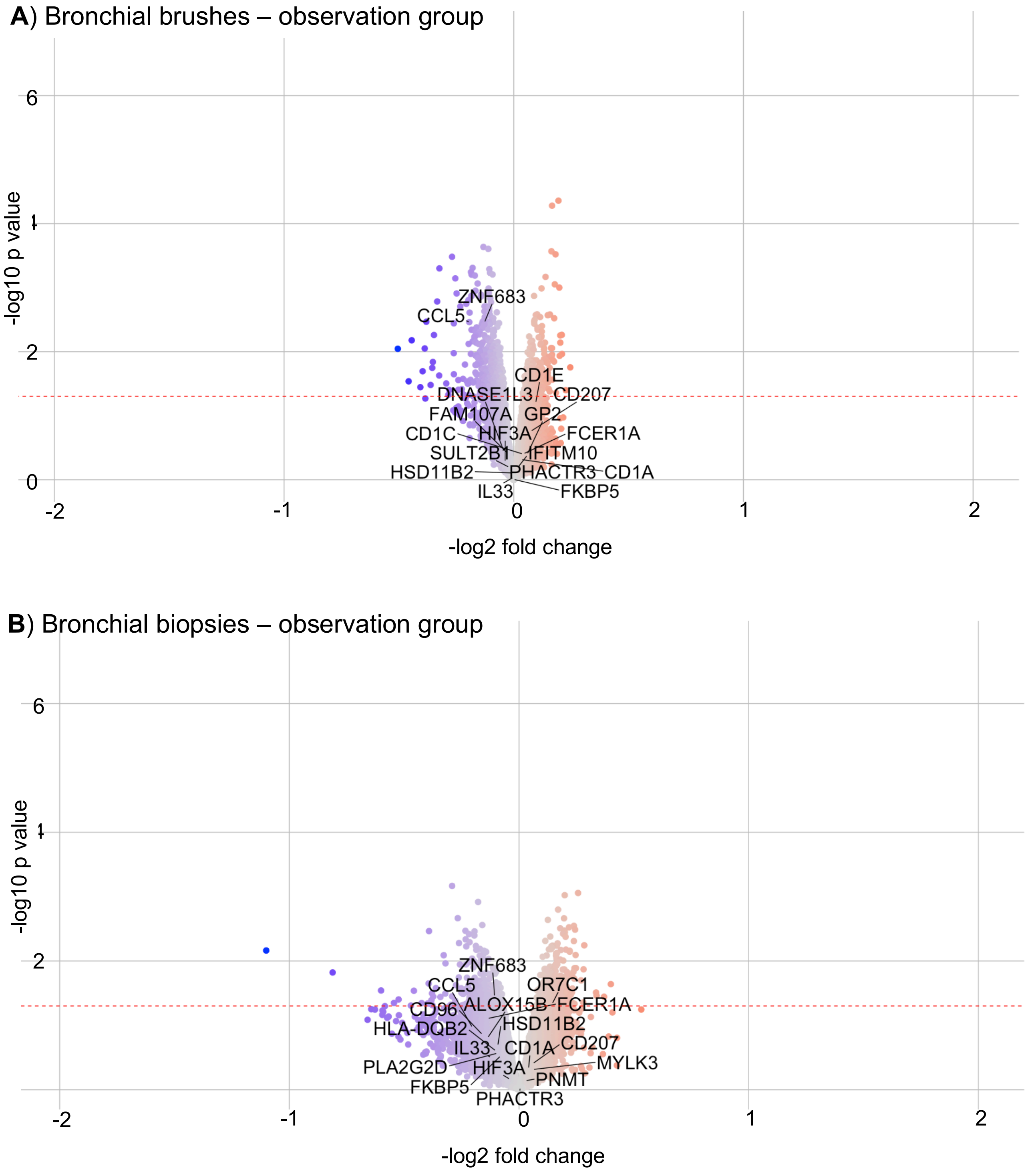

Supplementary Figure E7

Changes in gene expression measured by RNAseq following 4 weeks observation (control group). **A**) Bronchial brush volcano plot. **B**) Bronchial biopsy volcano plot. The labelled genes are a subset of those that were significantly changed by ICS. p values have not been adjusted.

Supplementary Figure E8

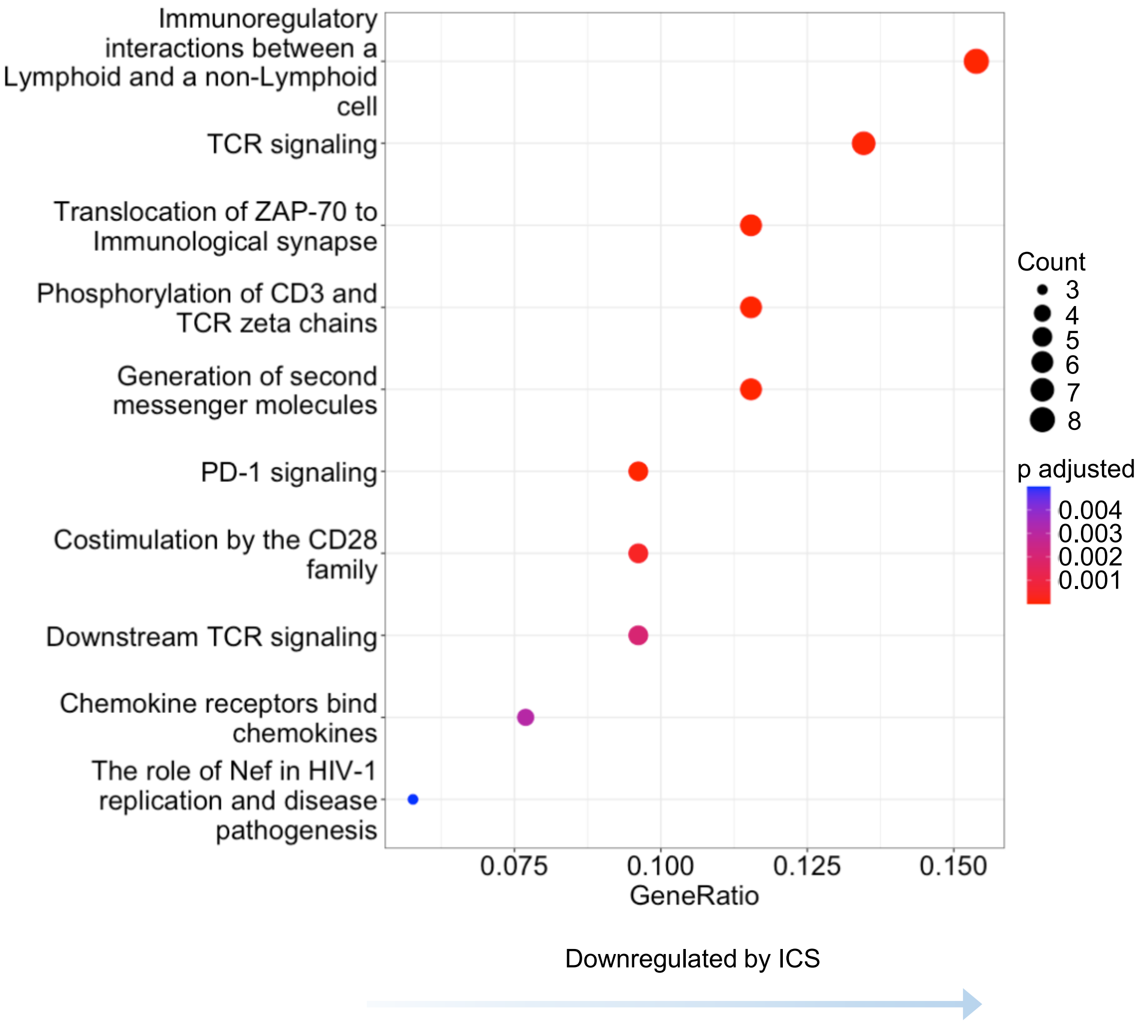

Supplementary Figure E8. Reactome pathway analysis of bronchial brushings

Reactome pathways significantly differentially enriched in bronchial brushing downregulated genes (log2 fold change >0, adjusted P value <0.25) after 4 weeks of inhaled fluticasone (treatment group only). x axis represents the gene ratio, the percentage of total differentially expressed genes in the given pathway; node size is proportional to number of genes differentially expressed in the pathway; node colour is proportional to Benjamini-Hochberg adjusted p value.

Supplementary Figure E9

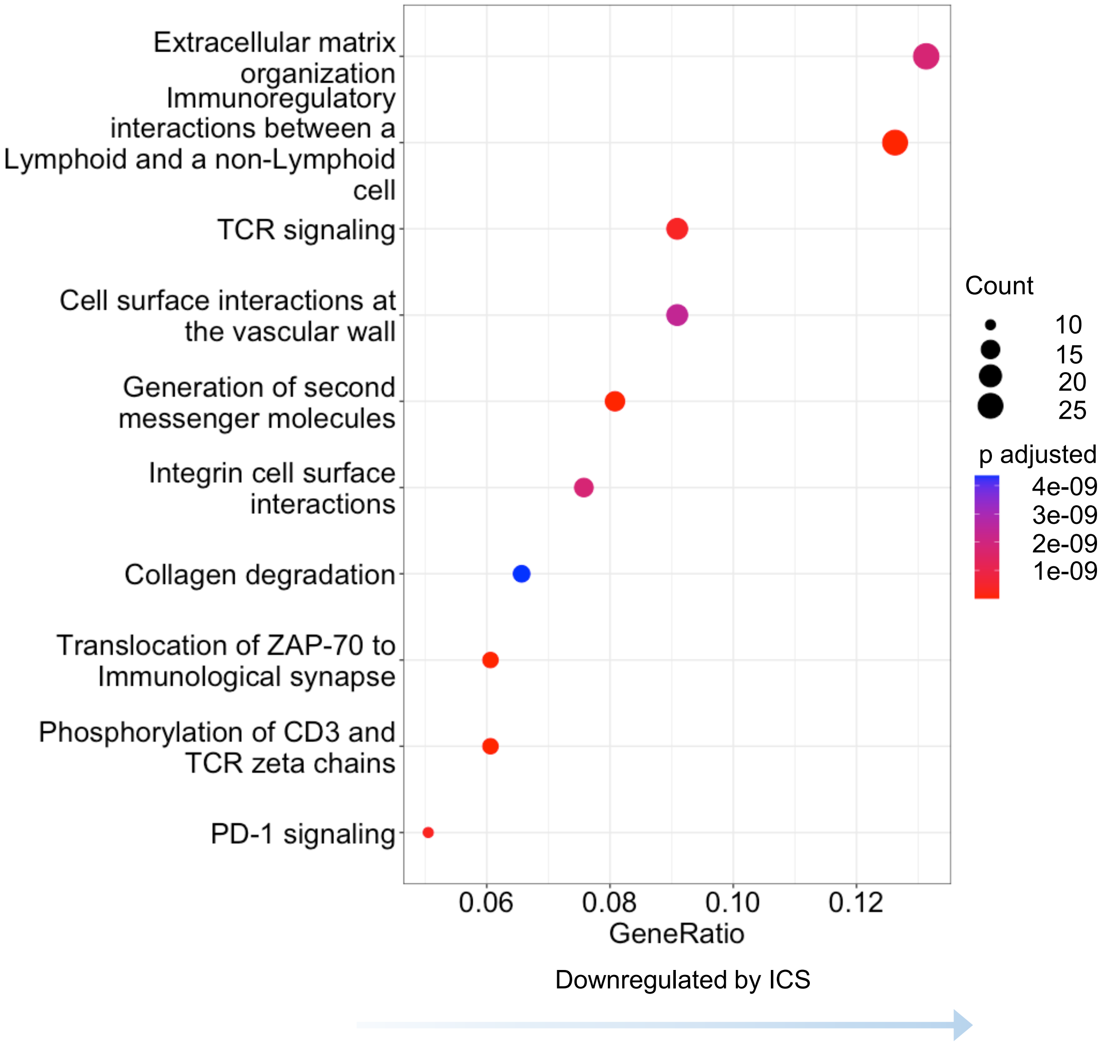

Supplementary Figure E9 Reactome pathway analysis of bronchial biopsies

Reactome pathways significantly differentially enriched in bronchial biopsy downregulated genes (log2 fold change >0, adjusted P value <0.25) after 4 weeks of inhaled fluticasone (treatment group only). x axis represents the gene ratio, the percentage of total differentially expressed genes in the given pathway; node size is proportional to number of genes differentially expressed in the pathway; node colour is proportional to Benjamini-Hochberg adjusted p value.

Supplementary Figure E10

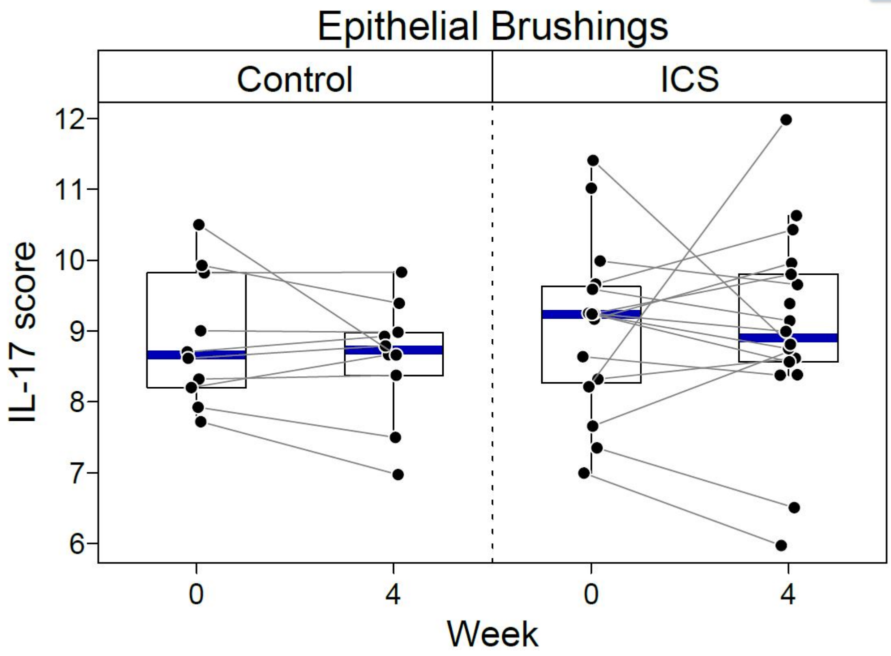

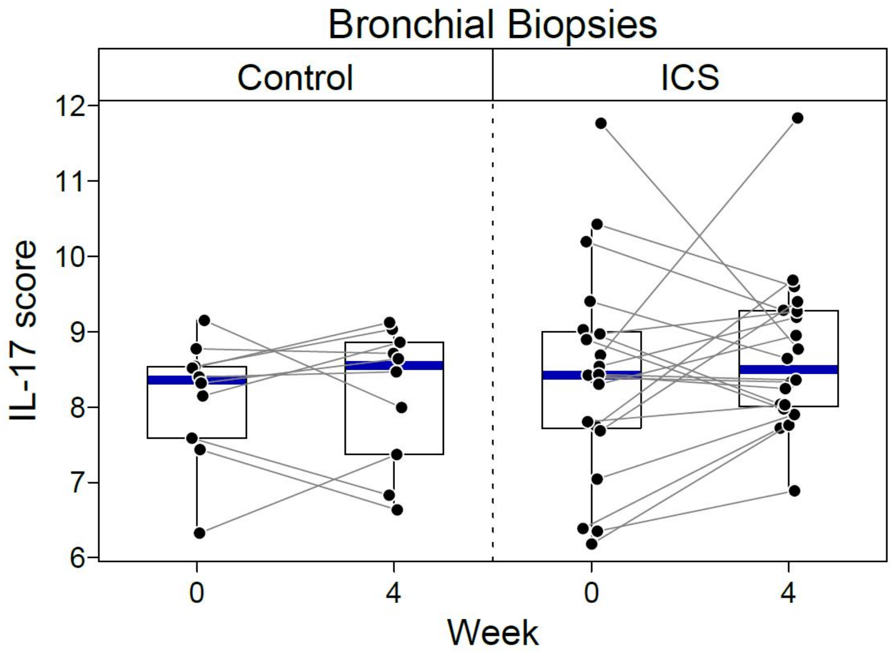

Supplementary Figure E10.

Expression of an IL-17-dependent gene signature in bronchial brushes and biopsies in response to 4 weeks treatment with inhaled fluticasone, or observation (control).

Supplementary Figure E11

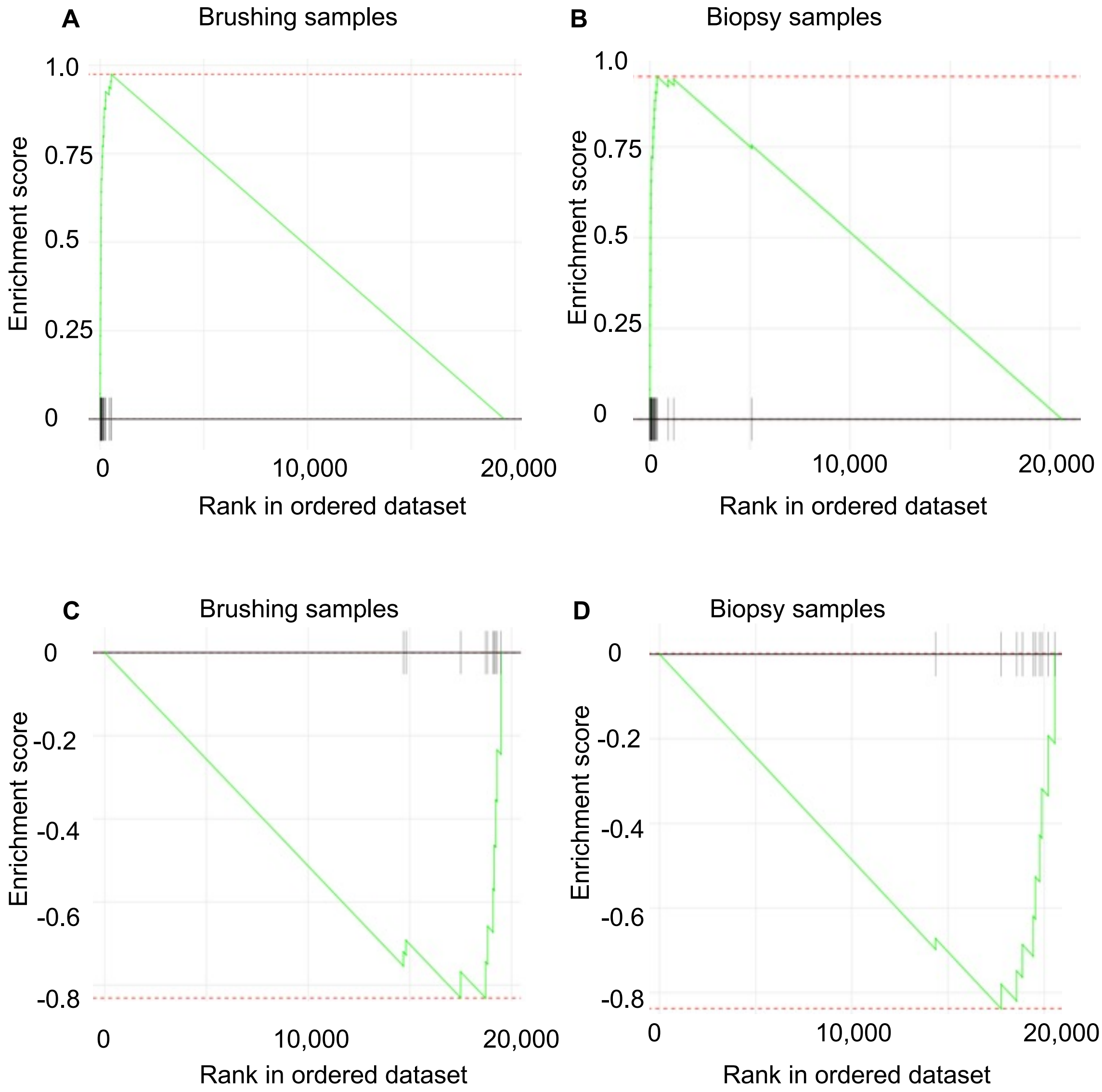

Supplementary Figure E11

Geneset enrichment analysis profiles amongst fluticasone treatment-related differentially expressed genes in healthy controls, showing very strong enrichment for a set of 26 genes shown to be induced (**A**, **B**) in participants with asthma by 10 weeks of inhaled fluticasone 500 mcg BD in a microarray analysis of epithelial brushings by Woodruff *et al* (E26). **B,D**) Enrichment of genes known to be down-regulated in Woodruff *et al* (E26). NES, normalised enrichment score. Adjusted P<<0.0001.

Supplementary Figure E12

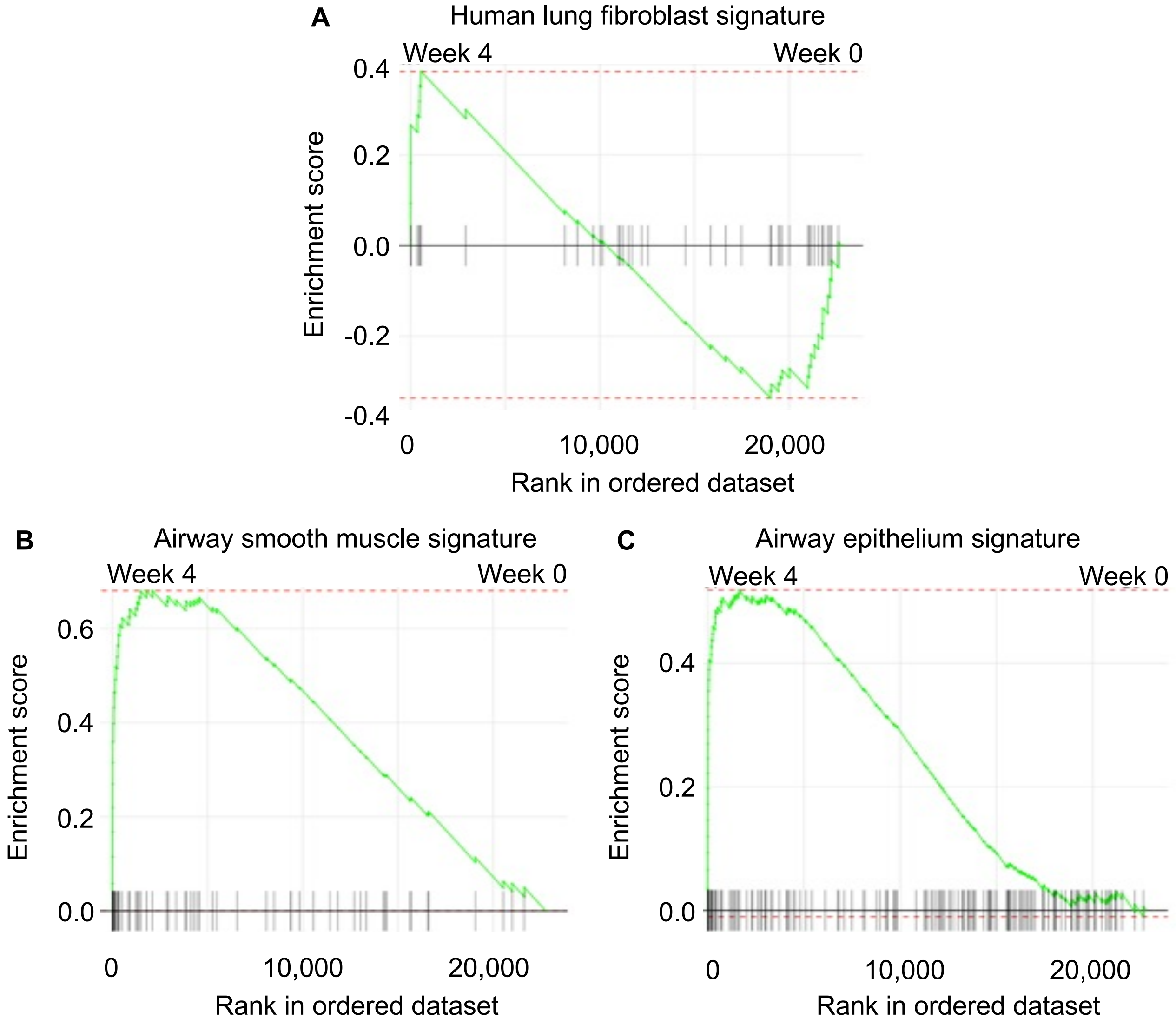

Supplementary Figure E12

Geneset enrichment analysis (GSEA) assessing enrichment profiles for genesets derived from structural cells, amongst fluticasone treatment-related differentially expressed genes in healthy controls. **A**) Genes induced with log fold change >3, adjusted P <0.05 in IMR-90 human lung fibroblasts treated with cortisol (E23). No enrichment observed, P>0.05. **B**) Genes induced with log fold change >3, adjusted P <0.05 in primary human airway smooth muscle cells treated with fluticasone propionate (E24). Geneset highly enriched, normalised enrichment score (NES) 2.7, adjusted P $<1x10^{-11}$. **C**) Genes induced with log fold change >1, adjusted P <0.05 in primary human bronchial epithelial cells treated with budesonide (E25). Geneset highly enriched, NES 2.5, adjusted P $<1x10^{-11}$.

Supplementary Figure E13

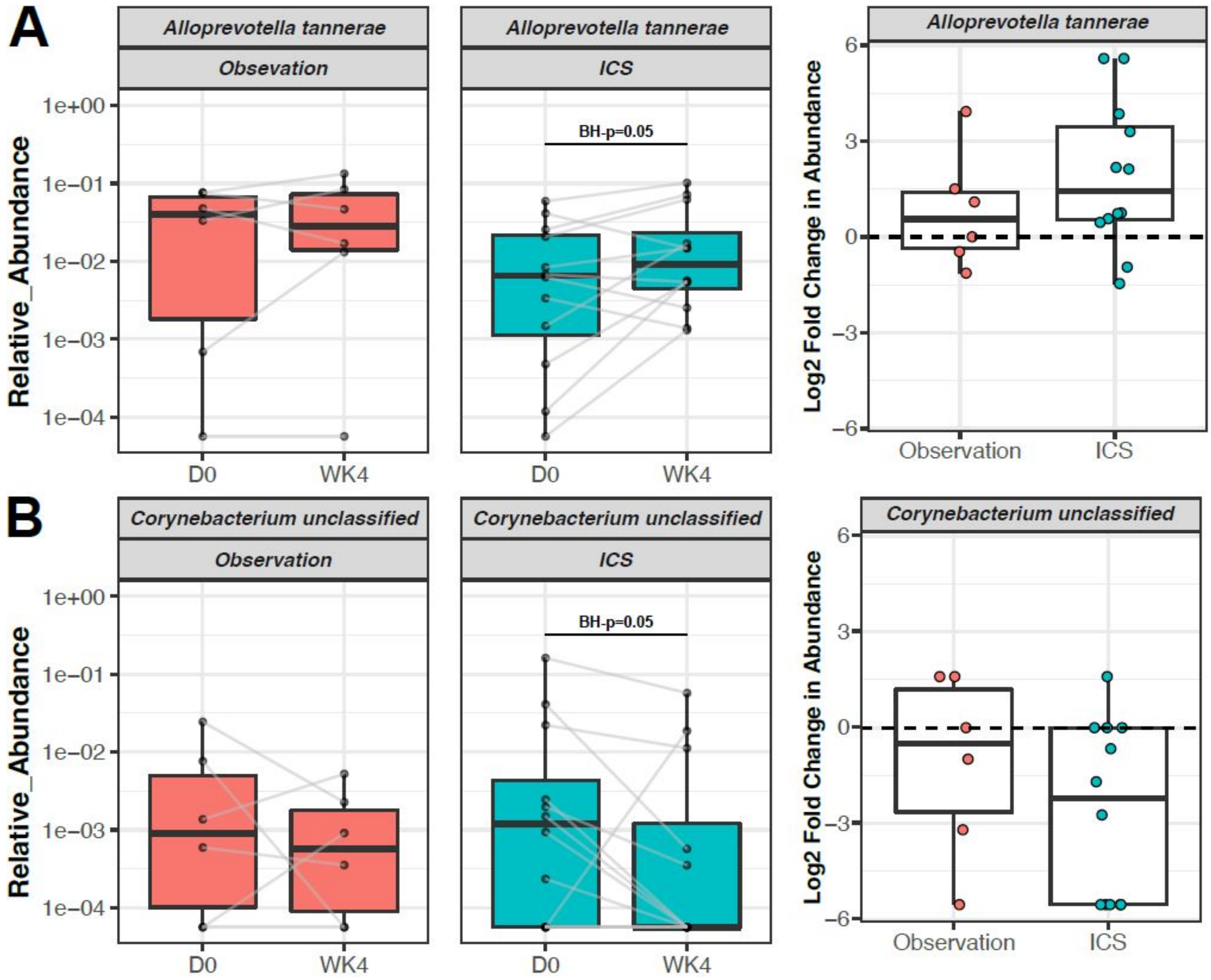

Supplementary Figure E13

Changes in **A**) Alloprevotella tannerae and **B**) Corynebacterium unclassified after 4 weeks of ICS-treatment or observation, which were significant with Benjamini-Hochberg adjusted p-value < 0.05, expressed as log2 fold change in relative abundance of taxa.

Supplementary Figure E14

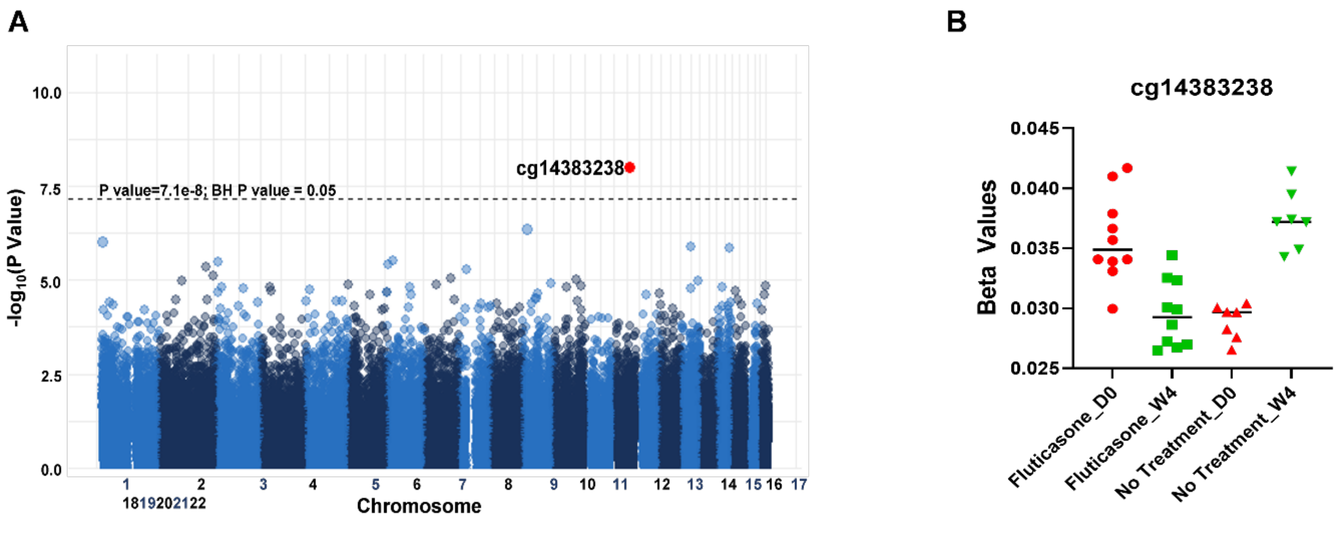

Supplementary Figure E14

A) Manhattan plot depicting the results of the linear regression interaction model investigating the effect of fluticasone treatment for 4 weeks. Genomic location of CpG sites are represented on x-axis and -log$_{10}$(p values) are represented on the y axis. The highlighted point (red) represents the only CpG site (cg14383238) that was significantly differentially methylated at week 4 compared to day 0 in response to fluticasone treatment. **B**) Contrasting trends observed in cg14383238 methylation between week four and day zero of fluticasone-treated and untreated groups. (D0- Day 0; W4- Week 4).
